# Flows of people in villages and large centres in Bronze Age Italy through strontium and oxygen isotopes

**DOI:** 10.1371/journal.pone.0209693

**Published:** 2019-01-09

**Authors:** Claudio Cavazzuti, Robin Skeates, Andrew R. Millard, Geoffrey Nowell, Joanne Peterkin, Marie Bernabò Brea, Andrea Cardarelli, Luciano Salzani

**Affiliations:** 1 Durham University, Department of Archaeology, Durham, United Kingdom; 2 Istituto Centrale per la Demoetnoantropologia, Rome, Italy; 3 Durham University, Department of Earth Science, Durham, United Kingdom; 4 Independent Researcher, Verona, Italy; 5 Università di Roma, ‘La Sapienza’, Dipartimento di Scienze dell’Antichità, Rome, Italy; New York State Museum, UNITED STATES

## Abstract

This study investigates to what extent Bronze Age societies in Northern Italy were permeable accepting and integrating non-local individuals, as well as importing a wide range of raw materials, commodities, and ideas from networks spanning continental Europe and the Mediterranean.

During the second millennium BC, the communities of Northern Italy engaged in a progressive stabilization of settlements, culminating in the large polities of the end of the Middle/beginning of the Late Bronze Age pivoted around large defended centres (the *Terramare*). Although a wide range of exotic archaeological materials indicates that the inhabitants of the Po plain increasingly took part in the networks of Continental European and the Eastern Mediterranean, we should not overlook the fact that the dynamics of interaction were also extremely active on local and regional levels.

Mobility patterns have been explored for three key-sites, spanning the Early to Late Bronze Age (1900–1100 BC), namely Sant’Eurosia, Casinalbo and Fondo Paviani, through strontium and oxygen isotope analysis on a large sample size (more than 100 individuals). The results, integrated with osteological and archaeological data, document for the first time in this area that movements of people occurred mostly within a territorial radius of 50 km, but also that larger nodes in the settlement system (such as Fondo Paviani) included individuals from more distant areas. This suggests that, from a demographic perspective, the process towards a more complex socio-political system in Bronze Age Northern Italy was triggered by a largely, but not completely, internal process, stemming from the dynamics of intra-polity networks and local/regional power relationships.

## Introduction

In recent years stable and radiogenic isotope studies have provided unprecedented insights into mobility of people in the past [1,2]. There has been an enormous increase of data and isotopic baselines for almost all regions of Europe, although with different intensities, methodologies and goals. Compared to other areas of the continent, Northern Italy has been only minimally covered by strontium and/or oxygen isotope investigations, with a specific focus on unique individuals (e.g. the Iceman) or single contexts, none covering the Bronze Age [3–5]. As we will argue below, these previous studies remain relevant for the present study, since they provide strontium and oxygen isotope data for several areas between the Alps, the Po and Adige River valleys and the Northern Apennine mountains.

The Bronze Age is commonly regarded as an extremely dynamic period of European prehistory. Mobility of people, raw materials (notably metal), goods and ideas contributed to the shaping of identities and the establishment of long distance networks that persisted into later centuries. Recent aDNA analyses seem to confirm this general picture, and also indicate that the current genetic variability of European populations was largely shaped during third and second millennium BC demographic expansions, migrations, admixtures and replacements [6–10]. Until now, only five genomes have been analysed from Northern Italy and all of them from Copper Age contexts (Remedello, the ‘Iceman’, and Parma-via Guidorossi). We still lack data to evaluate subsequent relations between the population dynamics and socio-political transformations that occurred in the area over the course of the second millennium BC. Nevertheless, it is clear that a crucial role was played by the Terramare culture in the Po Plain during the Middle and Recent/beginning of the Final Bronze Age (1650–1150 BC). The Terramare culture has consistently attracted the attention of European archaeologists, due to the exceptional preservation of its settlements, defensive and agricultural infrastructure, cemeteries, and other ritual sites, but also because the Terramare occupied a crucial location along the corridor that connected the palatial Mediterranean and continental European polities, especially those settled in the Danubian-Carpathian Basin [11]. The rise and the fall of the Terramare culture is consequently understood as closely tied to the process that involved the transformation of village communities in Northern Italy into a more articulated network based on ‘nodes’ and dependent communities, a political model possibly inspired by eastern precedents, but largely independent and locally interpreted, to judge by the isotopic evidence presented below.

Another distinctive feature of the Terramare culture is the widespread adoption of the ‘urnfield’ model as a funerary custom, probably around 1450 cal. BC, significantly earlier than the establishment of the ‘*Urnenfelderkultur*’ in other areas of Italy, Europe and the Mediterranean [12]. The transition from flat inhumation burial to cremation and internment in urns has been interpreted by archaeologists as reflecting a population shift due to the migration of ‘*Italici incineratori*’ (‘cremating Italians’) to Northern Italy from the Alpine/Danube areas [13–16]. Although this hypothesis has fallen out-of-fashion, individual and population mobility studies remain a fertile ground of research to test both old and new theories.

The goals of the present study relate to the above-mentioned issues: (1) to reconstruct individual mobility at three chronologically consecutive key-sites, which effectively embody three developmental stages in the process towards a more complex socio-political system and socio-economic structures; (2) to compare strontium and (whenever possible) oxygen and carbon isotope ratios on sample of human remains from inhumations, cremations, and bi-ritual cemeteries south and north of the Po River; and (3) to provide further strontium and oxygen local baselines to support future isotope studies.

## Principles of strontium and oxygen isotope analysis in human mobility studies and in Northern Italian isoscapes

Strontium and oxygen isotope ratios in odontoskeletal remains are regularly employed to assess the provenance and retrace the mobility of individuals in different stages of their lives. The combination of both isotopes’ composition has been frequently used in mobility studies (e.g. [17–21]), as it enables the combination of two parameters that depend on geology and climatology, food and drinking water.

The relationship between the different biological tissues and the isotopic composition of local environments varies with the type of element/isotope and tissue targeted by the analyses. The partitioning of isotopes in the environment depends on many factors, such as the geological nature of the substrate, environmental conditions, soil type, hydrological dynamics, amount of precipitation, plant species and their distribution [22,23].

Concerning strontium, the provenance of individuals is determined by comparing the ratio between strontium-87 (^87^Sr) and strontium-86 (^86^Sr) in bones/teeth with the local baseline values measured in faunal/vegetal samples (modern or ancient) from the site or its geologically coherent immediate hinterland. The technique has been in use for more than 30 years in bioarchaeological research and is described in detail in a number of publications [17, 24–33].

Radiogenic strontium-87 (^87^Sr) originates over time from the radioactive decay of rubidium-87 (^87^Rb; half-life of 48.8 Ma) and represents approximately 7% of the overall strontium contained in rocks. The other strontium isotope relevant to provenance studies, Strontium-86 (^86^Sr), is instead stable. Their ratio (^87^Sr/^86^Sr) is therefore dependent on the age of a given bedrock, but also on its geochemical nature. Older geological units (>100 Ma), such as Palaeozoic metamorphic and Mesozoic igneous rocks in the Alps, generally display higher ^87^Sr/^86^Sr values (≥0.71), while younger materials, such as Cenozoic marine carbonates and chalks in the Apennines, show lower ratios (≤0.709) (see also [21]). The sediments that form alluvial plains reflect the ratio of their parent material, or an admixture of the ratios that characterize the different geological units affected by the erosive activity of rivers in the uplands.

Soluble strontium then enters the food chain, first absorbed by plants and subsequently fixed in the bones and teeth of animals/humans by replacing calcium in the bioapatite fraction of the tissues.

Tooth enamel is the most common tissue targeted for strontium analyses on inhumations, as enamel forms during infancy, early childhood and adolescence, and does not remodel during the individual’s lifetime, consequently yielding the isotopic value of the origin of the food that was consumed in the various stages of life, from birth to early adulthood. For later prehistoric times in Europe, we can reasonably assume that most of the food consumed by communities was produced in the surroundings of settlement sites. In contrast to bones, tooth enamel has been proved to be less susceptible to diagenesis and contamination from the soil than bioapatite (e.g. [34–38]).

Concerning cremations, as tooth crowns tend to be destroyed by the heat, tooth enamel sampling is only very rarely a viable option. Bone instead survives to high temperatures, albeit deprived of the organic component between 350 and 600°C and modified in shape and in crystal size above 650–750°C, with the temperature causing the typical white ‘calcined’ appearance of the bone [39–41]. Strontium isotope composition in bone does not significantly change as a consequence of fire treatment [42]. All calcined bone preserves an *in vivo* signal, and indeed seems even more resistant to the small diagenetic alterations that affect enamel [43].

Out of all anatomical parts, the *pars petrosa* (petrous portion) of the temporal bone represents an effective target for strontium isotope analyses for a variety of reasons. It is the densest bone of the skeleton, and, therefore, frequently preserved in prehistoric urn cremations. It begins its formation *in utero* at approximately 16–18 gestational weeks and becomes fully ossified at the time of birth. Moreover, the otic capsule in the inner part of the petrous portion does not undergo any further remodelling after the age of 2 years [44–46]. Considering that the petrous portion forms primarily before the age of weaning, its strontium isotope ratio is assumed to reflect the origin of the food consumed by the woman who breastfed the infant. It also seems reasonable to assume that in the vast majority of cases the infant’s mother/wet nurse did not change over the time of breastfeeding, did not move separately, and did not consume foods of a different origin.

Overlapping of ^87^Sr/^86^Sr values across geographic regions with a high degree of geological formations may complicate the accurate assessment of provenance. The integration of a second provenance-based isotope, such as oxygen, can clarify some uncertain cases.

Oxygen isotope ratios (^18^O/^16^O, or δ^18^O) in unburnt human bones and teeth are related to the isotopic composition of local drinking water (both rain- and ground-water), which essentially depends on the latitude, altitude and atmospheric temperature [47]. Due to its lighter mass, H_2_^16^O requires less energy to evaporate, and evaporates preferentially, so in warm regions groundwater tends to be higher in ^18^O. Moreover, since water vapour condenses in the atmosphere to form rain, H_2_^18^O is preferentially incorporated in water droplets. Cold and high regions are therefore poorer in ^18^O as it precipitates out in lower latitudes/altitudes. The ratio between ^18^O and ^16^O is expressed with the delta (δ) notation as δ^18^O per mil (‰), where δ = *R*_*sa*_*/R*_*st*_–1, *R* being the isotopic ratio, ‘*sa’* the sample and ‘*st’* the reference standard (VSMOW) [48].

**Fig 1:**
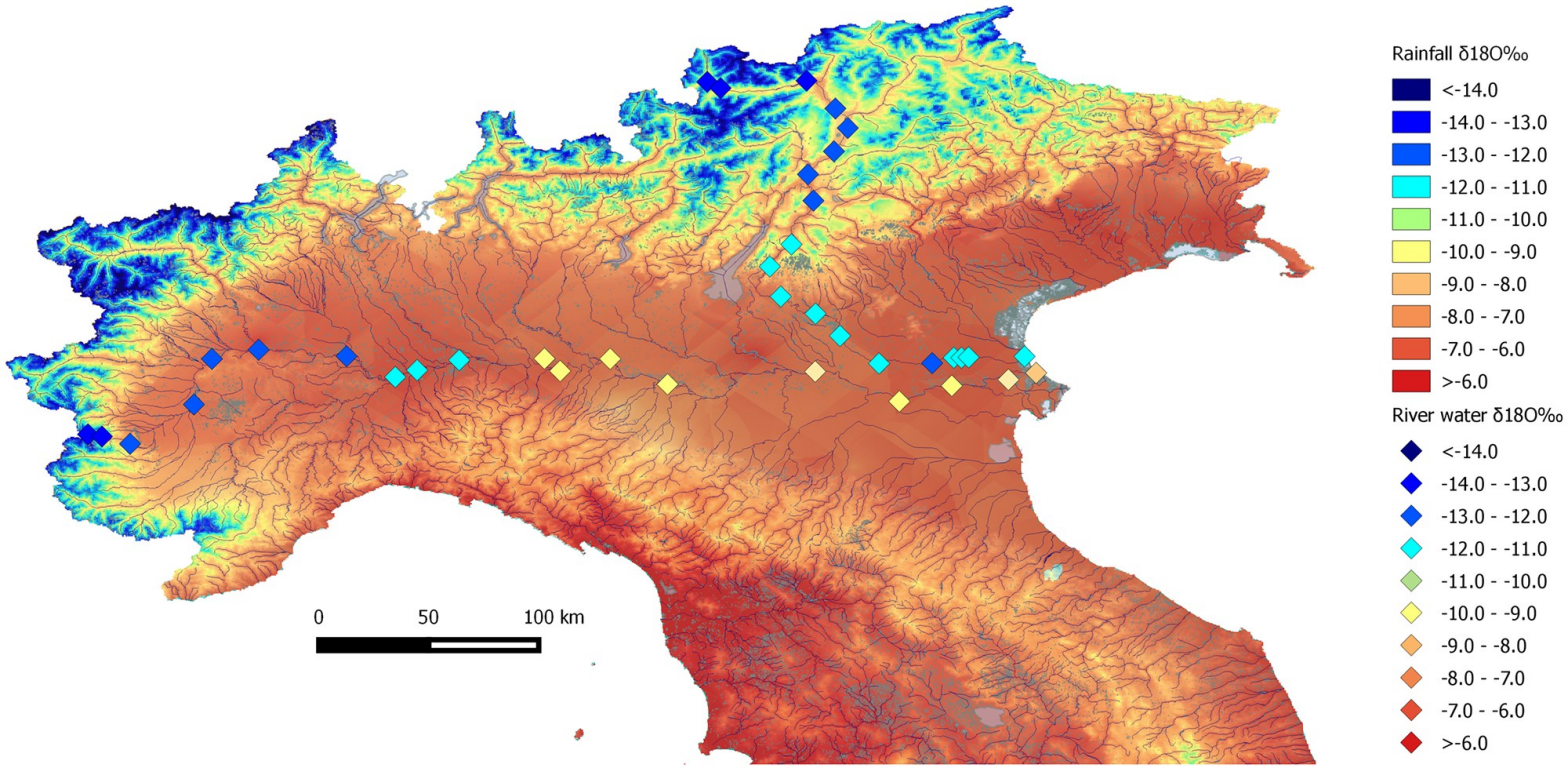
Rainfall and Po and Adige river water δ^18^O isoscape of Northern Italy. The map is constructed by using geospatial data from Giustini et al. [50] available at: https://www.sciencedirect.com/science/article/pii/S2214581816300143#ec-geospatial-data and plotting data from Marchina et al. [53] and Natali et al. [54].

Regarding Italy, the first ‘isoscape’ of oxygen isotope composition was constructed by Longinelli and Selmo [49]. Using samples of rainwater from different gauges along the peninsula and islands, they were able to define three Local Meteoric Water Lines for Northern, Central and Southern Italy. Subsequently, the dataset has developed rapidly and has been used in a variety of applications, from hydrogeology to ecology and, more recently, archaeology. Italian meteoric precipitation isotopes vary widely, according to four main factors: 1) the differing contribution of air masses from Northern Europe and the Atlantic Ocean against those from Africa and the Mediterranean Sea; 2) the latitudinal extension of Italy (between 35° and 47° N); 3) the narrow shape of the central part of the Italian peninsula; and 4) the physiography, ranging from sea level to 4000 m of altitude in the Alps [50,51]. This latter fact is particularly relevant as precipitation becomes increasingly depleted in ^18^O at higher altitude. The intensity of the ‘altitude effect’ in Northern Italy is therefore of primary importance in tracing mobility from mountain areas to the lowlands, where the archaeological sites considered in this study are located. Rainfall isotopic values are lower in the Alpine area (between -12‰ to -8‰) and significantly higher in the plain areas (between -8‰ and -6‰) [50] (Fig 1). Recent studies have suggested that δ^18^O measured on dentine apatite phosphates of archaeological animals, albeit indicative of altitude, are moderately relevant for provenance studies, since the inter-individual variability from the same site can reach peaks of 4‰ and can therefore be misleading for the determination of local baselines [52]. For these reasons, oxygen must be combined with strontium and, eventually, lead isotopes.

Since Bronze Age individuals could obtain their water supply from various reservoirs, the rainfall oxygen isoscape data need to be integrated with the available data from major watercourses. Systematic geochemical and isotopic investigations have been carried out on the surface waters from the two main rivers of Northern Italy (Po and Adige), as well as large lakes, such as Lake Garda and its tributaries. δ^18^O along the course of Po River varies from -13.1‰ at the higher altitudes to -8.5‰ in the delta [53], while the Adige River waters range from -13.8‰ to -10.9‰ [54]. The difference in the values at the delta between the two main rivers derives from the fact that the River Po, but not the Adige, receives a significant contribution of southern tributary waters and groundwater, with higher δ^18^O values. Lake Garda and its outflowing Mincio River instead show lower values (between -9.2‰ and -7.0‰), probably as a consequence of a greater contribution of rainfall to this large water basin [55].

Second permanent molar teeth are usually selected for oxygen isotope analysis, since they mineralize between 2–3 and 8 years of age [56], thus yielding the signature of the place of origin (or, more precisely, of childhood). First molars tend to be excluded for their likelihood of being characterized by breastfeeding isotopic signatures [57]. The comparison between local water and tooth enamel oxygen values allows non-local individuals to be distinguished, although the interpretation of the results is not always straightforward. Beside variations due to admixture of different groundwater/rainfall and watercourse sources, some individuals may have moved between warmer and cooler areas during childhood enamel formation. Food treatment can also contribute to the variability: boiling, brewing and cooking practices all produce changes in the typical isotopic values of fresh food and drinks from a certain area [58,59].

The best approach is to limit direct or narrow comparisons between local water and bio-phosphate or carbonate δ^18^O values [60,61], and instead to analyse the intra-population statistical distribution and assess a local range, outside of which non-local individuals should be positioned. The assumption is that the majority of individuals had a similar drinking behaviour, which should result in a rather homogeneous isotopic outcome [59].

A general comparison between human values and drinking water ‘isoscapes’ can be nonetheless indicative. This procedure involves measuring δ^18^O from phosphates [62] and from carbonates [61,63] of the hydroxyapatite.

To estimate the oxygen isotopic composition of ancient drinking water from enamel carbonate data we use the equation of Chenery et al. [61]: δ^18^O_Drinking water_ = 1.590 x δ^18^O_VSMOW(carbonate)_− 48.634, although such comparisons must be undertaken with a certain caution [20]. The bivariate analysis with ^87^Sr/^86^Sr, however, considers the δ^18^O_VSMOW(carbonate)_.

## Geology of Northern Italy and biologically available strontium baselines (BASr)

**Fig 2:**
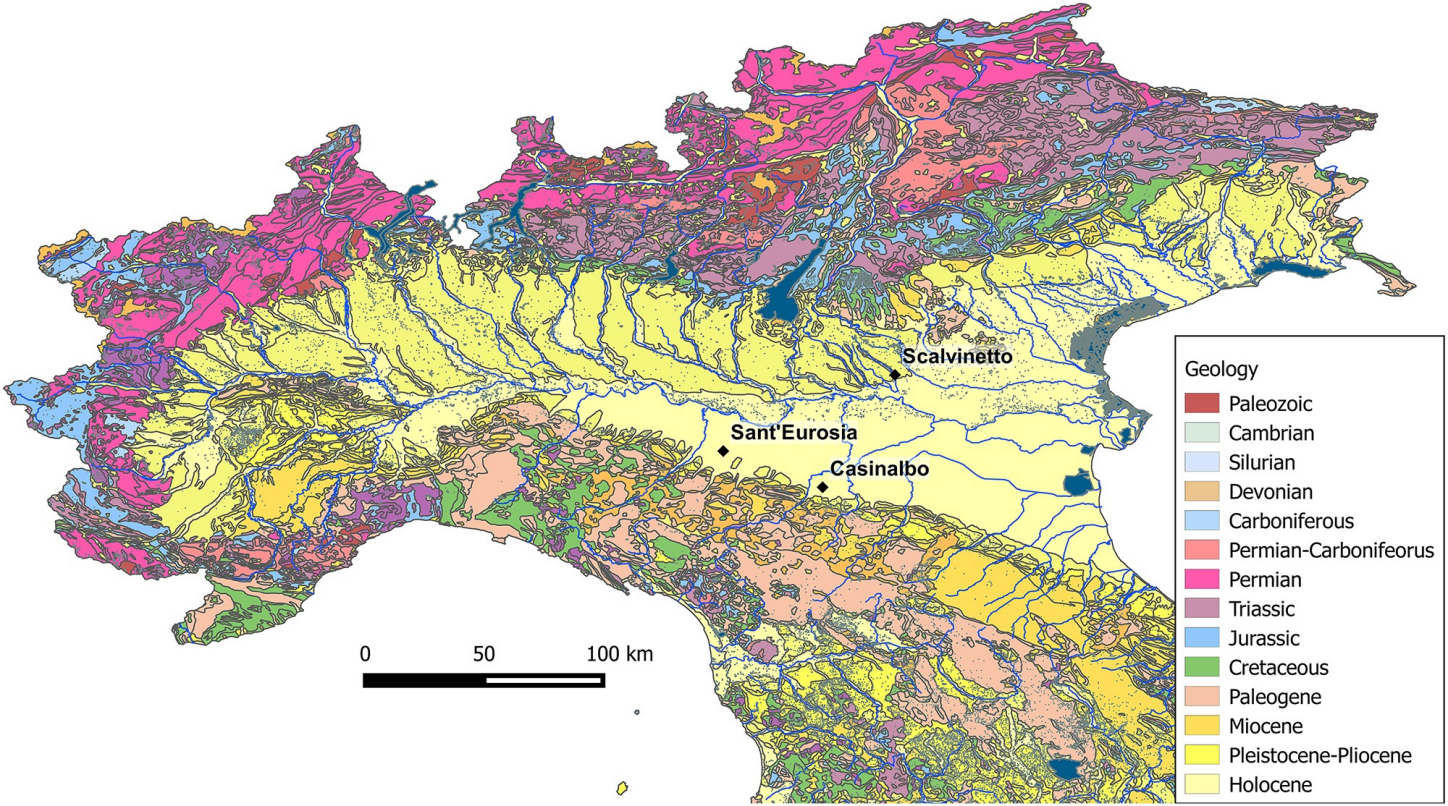
Geology of Northern Italy and location of the analysed archaeological sites. The map is constructed by using public domain wms data downloadable from http://wms.pcn.minambiente.it/ogc?map=/ms_ogc/WMS_v1.3/Vettoriali/Carta_geolitologica.map under a CC BY license, with permission from Geoportale Nazionale.

Northern Italy might be regarded as an enormous amphitheatre with the Alps forming two-thirds of the terraces in the north and the west, and the Apennines one third in the south, while the arena is the large alluvial plain, mainly formed by the River Po and its tributaries, with the Adriatic Sea as the background (Fig 2).

High structural and morphological relief and exposure of deep crustal rocks characterize the Alps, which show a variety of geological formations, mainly pre-Cretaceous [64]. In the Central and Eastern Alpine area, several major coherent units or associations are relevant to the present study: Permian volcanics/granites and Mesozoic carbonates in the Upper Adige and Isarco River valleys (Alto Adige); Triassic and Jurassic dolomites on both sides of the Adige River (Trentino); Carboniferous-Permian phillites on the left bank of the Adige (Trentino); Jurassic-Cretaceous limestones and dolomites with flint, radiolarites and marls (Monti Lessini, Veneto); and glacial deposits in the Lake Garda morainic amphitheatre (Veneto). In the Veneto region, between the Adige and Brenta Rivers, Cenozoic limestones, marls and turbidites, as well as rhyolitic rocks, outcrop in the upland areas of Colli Berici and Colli Euganei (see maps of Servizio Geologico d’Italia).

The Apennines are largely characterized by lower structural and morphological relief, mainly composed of Cenozoic carbonate series, turbidites, marls, limestones and flysch, with sporadic outcrops of Miocene chalks. Some older Cretaceous-Jurassic igneous ophiolites also sporadically appear in the western part, especially west of the Taro River.

The River Po drains the water flowing from the two mountain chains and marks a north/south discontinuity in terms of geological composition of the soils. Alpine-derived materials, deposited by the left-bank tributaries, form the northern alluvial plain, while Apennine-derived materials, deposited by right-bank tributaries, form the southern alluvial plain. Other rivers of significant water volume flow directly to the Adriatic Sea, such as the Adige and Reno, respectively north and south of the River Po.

The sites selected for this isotopic investigation are situated in the two main parts of the plain, one in the north (Scalvinetto/Fondo Paviani) and two in the south (Sant’Eurosia and Casinalbo). Due to the above-mentioned geological dynamics, we can expect significantly different local isotopic signatures.

Ten different geolithological zones have been identified for facilitating the mapping of the strontium isoscape, and ultimately to assess the provenance of buried individuals (S1 Fig and S1 and S2 Tables).

Thirty-five new baseline values have been produced within the present study, analysing animal tooth enamel from the sites (whenever possible) or modern snails found on targeted geolithological units at different distances. Ancient faunal remains have been considered to represent a robust average bioavailable Sr isotope composition over their feeding area [22,28]. However, it is very unlikely that humans and domestic animals ate food from distinct locations, marked by different isotope compositions.

As Tafuri et al.’s recent work has indeed demonstrated for the *Terramara* at Fondo Paviani (as well as for other *Terramare* sites) that cattle, sheep/goats and domestic pigs were fed with C_4_ plants, presumably millet [65], which was also identified in the pollen series and phytolith record from the site [66]. This means that, during the Terramare period, animals were almost certainly fed with fodder cultivated in the surrounding fields, and for this reason, their strontium isotope composition presumably reflects the local baseline. Obviously, animals could also be part of gifts/exchanges with other distant communities and, therefore, this source has to be considered critically in comparison with other sources, but aids in validating the inferred bioavailable ranges. For our study, we have added snail shells, also used by several authors as indicator of locally bioavailable strontium source [21,31,67–73]. Some authors have pointed out that land snail shell ^87^Sr/^86^Sr can be biased towards values for soil carbonates; nonetheless their values are usually close to ground vegetation [74].

Food chemistry represents another source of BASr, especially with regards to wine ‘authentication’ or geographic traceability, both north and south of the River Po [75–78].

We have also taken into account geological/paleontological studies from the Apennines, which analysed bedrocks [79,80], in addition to chemical analyses of natural mineral waters [81]. The work by Voerkelius et al. is relevant for comparison with the nearest baselines, but strontium isotope ratios from spring waters can only be used with caution, as they represent a very locally-specific kind of evidence, while an individual’s diet is an admixture of different sources from a specific, but wider, area.

Unfortunately, the Po plain is one of the most intensely exploited regions of Europe, with extremely few uncultivated, non-urbanized areas. A very recent detailed Sr isotope survey in Poland [82,83] showed that the modern biosphere (animals) and hydrosphere (surface waters) can be contaminated by anthropogenic strontium derived from agriculture, industrial and municipal sources. For that reason, comparison of multiple sample types is necessary to achieve a robust isoscape. Following Emery et al.’s ‘first map’ [21], we have considered previous studies, in order to make a comparison between four different sources, namely ancient animals, modern snail shells, modern plants and (whenever possible) bedrock. While each sample type has the possibility of a bias away from the values bioavailable to humans, these biases are unlikely to be in the same direction nor to be equal. Thus when these different sample types agree we can infer that the biases are negligible, and when they disagree any estimated local range will likely be too large, therefore reducing the number of inferred migrants.

The new data are integrated with a database of 199 ^87^Sr/^86^Sr values gathered from the existing literature (S1 Table), which contributed to the first published map of ^87^Sr/^86^Sr baselines for the whole Italian territory [21]. This map was inspired by a number of examples, all of them interpolating a variety of strontium sources, such as human and faunal data, modern plants, beef, cheese, sediment, soils, natural spring water, fossil, and tomato sauce [69,73,74,84–86]. Compared to other ‘isoscapes’, the strontium isotope map of Italy still lacks in spatial resolution and critical assessment of baselines, which certainly still need to be enhanced, but the materials used are not out of line with other published isoscapes which are widely used for interpretation of archaeological samples.

The variation of the currently available strontium isotope ratios for each of the ten geolithological zones is shown in S2 Table.

## Previous studies of human mobility in Northern Italy

Previous archaeological studies in Northern Italy have also provided local isotopic baselines, as well as an analysis of mobility for different periods, from the Copper Age (Iceman) to the Iron Age (Celts) and Early Medieval period (Lombards).

The first important results on strontium and oxygen isotopes were produced 15 years ago on the teeth of the Iceman and environmental samples from the upper Adige and Isarco river valleys. The mountains around the two rivers are characterized by Mesozoic impure carbonates with strontium isotope ratios ranging between 0.710 and 0.714, Permian volcanics between 0.717 and 0.719 and Carboniferous phyllites/gneisses between 0.720 and 0.725. Oxygen isotope ratios (VSMOW) of the river waters vary between -15‰ and -9.2‰ [3]. The ^87^Sr/^86^Sr of the Iceman’s tooth enamel ranges from 0.7198 to 0.7203 and δ^18^O on enamel carbonate between -10.98‰ and -10.56‰.

The study recently carried out in the fifth to eight century AD cemetery at Povegliano Veronese (Verona province) shows that the local Lombards’ strontium isotope ratios range from 0.7084 to 0.7091, while the soil samples from the same site range from 0.7082 to 0.7089 [5]. These results show a good overlap with snail samples from Nogara (Verona plain; 0.7084–0.7094), while other nearby baseline values display small but significant variations, such as in the areas around Scalvinetto/Fondo Paviani (0.7096–0.7098), Monti Lessini (0.7076–0.7083), and the moraine deposits of Lake Garda (0.7079–0.7080). The variability of BASr in the Verona province appears rather wide, considering the relatively small extent of this territory (approximately 3000 km^2^). Such a broad spectrum derives from the diverse geological formations, and the distinct fluvial basins, eroding and depositing sediments of different geological origin.

Compared to the baselines obtained for the territory between the Alps and the River Po, those registered for the area around the Celtic cemetery at Monte Bibele (Bologna province, Idice River valley) south of the Po River are significantly lower, ranging between 0.7089 and 0.7091 [4]. Even though the Idice valley is around 50 km east of Casinalbo and around 100 km east of Sant’Eurosia, the geology of the entire Emilia Romagna region is rather similar. In fact, baselines at Casinalbo and in its hinterland range between 0.7084 and 0.7090, and at Sant’Eurosia between 0.7082 and 0.7092. The range of baselines in Emilia Romagna (Apennine mountains and plain) should therefore range from 0.7080 and 0.7093, when also considering the spring waters analysed by Voerkelius et al. [81].

## Materials

A total of 104 buried individuals were sampled: 50 inhumed and 54 cremated.

For inhumations, we sampled enamel from second molars (M2), whose crown develops between 3 and 8 years of age and, when no M2 was preserved, from second premolars (P4), which are characterized by a similar pattern of growth [56]. First molars, having a more precocious development, were excluded, as their oxygen isotope composition can be significantly affected by breastfeeding [57]. For cremated remains we sampled the petrous portion, which forms at 0–2 years of age. This sampling strategy maximised the information obtained but the differing formation times need to be borne in mind when examining the results.

From the Early Bronze Age cemetery at Sant’Eurosia, we sampled all the excavated individuals with preserved dental elements. Pieces of enamel from 18 second molars (M2) and 2 second premolars (P4) were taken from 12 adult males, 4 adult females and 4 children (between 5 and 9 years of age). Sex and age were determined through the methods collected by several authors [87–90].

From the Middle and Recent Bronze Age urnfield at Casinalbo, we sampled 24 cremated individuals, comprising 9 adult males, 11 young or adult females, and 4 children (between 3 and 6 years of age) [91]. In one individual, t. 491, we sampled both a fragment of the petrous portion and a piece of cremated M2, in order to compare and test the homogeneity of the results.

From the Middle and Recent/Final Bronze Age bi-ritual cemetery at Scalvinetto, we sampled 60 individuals: 30 inhumed and 30 cremated. Among the inhumations, we sampled 8 adult males, 18 adult females and 4 subadults (between 5 and 18 years of age). Of three individuals (tt. 246, 313 and 405), samples from two different molars were taken for tracing possible movements between childhood and adolescence (M2-M3). Among cremations, we sampled fragments of petrous portions from 12 adult males, 11 adult females, 1 adult of undetermined sex, and 6 subadults of different age classes (‘*Infans 1*’ = 0–6 years of age; ‘*Infans 2*’ = 7–12 years of age; ‘*Juvenis*’ = 13–20 years of age). In the case of t. 58, an *Infans 1* individual, a piece of non-erupted M2 crown was sampled instead of petrous portions.

All sampled teeth and cremated bone fragments were part of the anthropological collection preserved at the Museo delle Civiltà di Roma (piazzale Marconi 14, Roma). Dr. Luca Bondioli, head of the Sezione di Bioarcheologia of the museum, provided the permission needed for this study.

The primary materials for our strontium baseline study come from faunal remains found at the archaeological sites and modern snail shell from various geolithological units located at different distances from the settlements.

Thirty-five new baseline values have been produced in the present study, thus enlarging the dataset of 199 BASr values reported in literature from the North Italian regions of Emilia Romagna, Veneto and Lombardy, also used by Emery et al. in their first ^87^Sr/^86^Sr isoscape of Italy [21].

## Site description

### Sant’Eurosia (Parma)

The Early Bronze Age (EBA) site at Sant’Eurosia is located on the southern margin of the Po plain, along the Baganza River, around 19 km south of the River Po and 10 km north of the first hills [92]. The recent discovery of 8 tumuli (2008) of a variable diameter, between 8 and 26 metres, was due to a rescue excavation on the south-eastern periphery of Parma (S2 Fig). The central core of each tumulus was always occupied by a single inhumation, oriented NW-SE (with variations of some degrees from case to case), while the surrounding ditches hosted a variable number of burials. Moreover, flat inhumations were found 12 metres south-east from the group of tumuli. Two of them are double burials, with the two skeletons arranged in a head-to-toe position. Ceramic and bronze materials can be attributed to an advanced phase of the end of the EBA1/beginning of the EBA2, dated to the 19^th^ century BC. The overall number of inhumations is 32: 12 adult males, 10 adult females, 1 juvenile, 5 children (5–9 years) and 4 infants (0–4 years). The location of the related settlement remains unknown.

### Casinalbo (Modena)

Casinalbo is the best-known cemetery in the area of the Bronze Age *terramare* settlements south of the River Po (S3 Fig). An extensive and detailed monograph has recently been published, providing archaeological data and osteological analysis for 349 burials [12,91]. Considering that urn cremation is the exclusive rite for all 673 excavated burials, it seems that Casinalbo adopted the model of ‘urnfield’ quite radically, although the chronology of the cemetery precedes the BzD *Urnenfelderkultur* in Northern Italy by approximately one century. According to chronotypology, stratigraphy and radiocarbon dates, the earliest burials date back to the Middle Bronze Age 2B-3A (c. 1450 BC) and the latest to the Recent Bronze Age 2 (c. 1150 BC) [93]. The three centuries of occupation end at the same time as the general collapse of the Emilian *Terramare* system, during the 12^th^ century BC [11]. The overall sex ratio among adults is very close to 1:1 [91]. This general picture is also reflected within the different burial groups, where generally an equal number of adult males and females is also represented. The percentage of individuals under 20 years is 33.2%. Among these subadults, those under 2 years are absent, as in other Terramare urnfields and also generally in Late Bronze Age Italy [94].

The Casinalbo site comprises both the funerary area and the settlement (*terramara*), located 200 m to north-east. This latter is relatively small, although it grew over the course of its occupation, probably at the end of MBA3 (from 1.5 to 2.5 ha), and was subsequently abandoned at the end of the Recent Bronze Age (12^th^ century BC). It essentially remained a centre of minor size compared to the large *terramare* of 15–20 hectares like Fondo Paviani (see below), though both were provided with typical defensive structures.

### Fondo Paviani/Scalvinetto (Verona)

Scalvinetto is one the largest bi-ritual cemeteries of the *Terramare* culture north of the River Po (S4 Fig). Its related settlement, the *terramara* at Fondo Paviani, is located 400 metres to the south-east, along the Menago River, an advantageous natural communication route between the Alps and the Po Plain. Recent investigation of the settlement site has better clarified the key role of Fondo Paviani in the so-called ‘Valli Grandi Veronesi’ geographical area during the transition from the *Terramare* system to a new political-territorial unit centred on Frattesina ([95] and references therein).

The occupation of Fondo Paviani spans the Middle Bronze Age 3 to the Final Bronze Age 1–2 (c. 1450–1000 BC). Like other *terramare* sites, after an initial phase during which the settlement was relatively small and less structured (around 7 ha), the central phases saw a substantial enlargement (up to 15–20 hectares) and a massive expansion of the defensive structures, namely the quadrangular ditch and embankment. This makes Fondo Paviani one of the largest fortified settlements in the Po plain and, as some authors have pointed out, it likely represents a node of a local polity.

It is intriguing to see the growth of this site in the light of the import of commodities, especially metal and amber, as well as the local production of ‘Appenninica’, Aegean-Mycenaean and Levantine-Cypriot Bichrome style pottery. The few available analyses of metal objects suggest that copper came to Fondo Paviani from the Valsugana (Trentino) sources to the north, probably via the Adige Valley, while amber has been identified as succinite and hence of Baltic origin [96,97]. While this evidence emphasizes contacts towards the Alps and the north of Europe, the two ‘exotic ceramic types are suggestive of the involvement of Fondo Paviani in a wider Mediterranean network [98,99]. Interestingly, the clay used for the production of the Aegean-Mycenaean and Levantine-Cypriot Bichrome style pottery has been determined as local, meaning that know-how and even artisans of Mediterranean/Oriental origin might have moved to the Po plain, as a consequence of existing socio-economic relationships between these poles [98].

All this new evidence suggests that Fondo Paviani took advantage of its strategic position between the Adige and the Po valleys, attracting raw materials, commodities, know-how and, above all, people both from the environs and from distant places. This is what is expected of a ‘central place’, as Michele Cupitò and Giovanni Leonardi have defined it, i.e. an emerging centre that broke with the long-lasting traditional socio-political system and triggered the process that led towards a more hierarchical socio-economic organization [95,100].

The Scalvinetto cemetery was discovered in 1989 and excavated between 1991 and 2001 [101,102]. 705 burials were recovered, 437 of which are urn cremations and 268 inhumations, all of them dated to within the same time-span of the settlement. The cremation graves are generally less deep than the inhumations, and some of the urn pits cut the inhumation graves. Looking at the horizontal distribution of the burials, it is evident that inhumations occupy the western part of the cemetery and the ‘urnfield’ the eastern area.

## Methods

### Strontium isotopes

Initial sample preparation was conducted in the Sezione di Bioarcheologia of the Museo delle Civiltà in Rome. Each tooth and petrous portion were sectioned using a flexible diamond impregnated cutting disc. The enamel was abraded from the surface with a dental burr and the removed material discarded. Any adhering dentine was then removed and a resulting clean core enamel of 20 mg isolated for oxygen and strontium isotope analysis. Cremated petrous portions were sampled using the method reported by Jørkov et al. [103]: the petrous bone was drilled at a 90° angle into the otic capsule (0.5–0.8 cm of depth), between the internal acoustic meatus and the subarcuate fossa using a low speed (2-mm diameter) drill, and a small fragment of bone was detached. Following Snoeck et al.’s method, samples were then pre-treated with 1M acetic acid for 3 minutes in an ultrasonic bath, followed by three rinses with with milliQ water and 10 min ultrasonication [104].

Human and environmental samples were prepared for strontium isotope analysis following previously published procedures [105,106]. They were dissolved in 16M HNO_3_, dried down and redissolved in 3M HNO_3_. The samples were loaded on columns of Eichrom Sr-spec resin, strontium was eluted with water and acidified to 3% HNO_3_ for analysis. All reagents were Teflon distilled and ultrapure Milli-Q system water was used.

Strontium isotope ratios were measured using a ThermoFinnigan Multi-collector ICP Mass Spectrometer (MC-ICP-MS) in the Northern Centre for Isotopic and Elemental Tracing at Durham University. Reproducibility of the standard NBS987 during analysis of samples was 0.710267 ± 0.000014 (2σ, n = 57). All values have been normalised to the accepted value of 0.710240 for NBS987.

### Methods-oxygen

Following Pellegrini and Snoeck’s recommendations, no chemical pre-treatment of the enamel was carried out, as it has been proven that the use of different procedures “introduces unpredictable often significant effects on the pristine isotopic composition” [107].

The δ^13^C and δ^18^O was measured in the carbonate (CO_3_) component of tooth enamel following the previous published procedures of Bentley et al. [108]. Approximately 2 mg of each powdered sample was reacted with 99% ortho-phosphoric acid for 2 hours at 70°C. The resultant gas mix of helium and CO_2_ was then separated and analysed via a Thermo Fisher Scientific Gasbench II interfaced with a Thermo Fisher Scientific MAT 253 gas source mass spectrometer for isotopic analysis. Duplicate analysis of 7 samples yielded a precision with a mean difference of 0.1‰ (1 s.d.) for δ^13^C and 0.13‰ (1 s.d.) for δ^18^O. Repeated analysis of both international reference material (NBS 19 n = 3, IAEA-CO-1 n = 3, LSVEC n = 3) and internal laboratory standards (DCS01 n = 7, Dobbins n = 2) yielded analytical reproducibility to be better than 0.04‰ (1 s.d.) for δ^13^C and 0.07‰ (1 s.d.) for δ^18^O. All values have been normalised to the accepted values of +2.49‰_VPDB_ and -46.6‰_VPDB_ for δ^13^C, and -2.40‰_VPDB_ and -26.70‰_VPDB_ for δ^18^O, for both IAEA-CO-1 and LSVEC.

### Locals vs non-locals?

Our method for assessing the provenance of the sampled individuals is designed to overcome, or at least mitigate, the sharp dichotomy between ‘locals’ and ‘non-locals’, which can to some extent be misleading or ambiguous, since such categories depend on the historical or cultural context we are analysing. The meaning of absolute distances, for example, varies across different physical landscapes and according to different modes of transport. The concept of space is therefore relative as is human mobility itself [109,110].

Commonly, an individual is considered ‘local’ if his or her strontium isotope ratio falls within the ‘local’ baseline range, or ‘non-local’ if the value falls outside. It is not always clear within what radius around the site the term ‘local’ is defined.

Given that different, even distant, places can be geologically (and therefore isotopically) similar, a straightforward approach might underestimate the number of outsiders. Theoretically, an incidence of ‘exotic’ food might also have an impact on isotopic ratios. It seems unlikely, though, that Bronze Age communities in Northern Italy traded in staple food, considering the high production capacity reached by intensive agriculture [11,111]. More plausibly, in this historical phase, strontium isotope ratios reflect the movement of people and not of the vast majority of the ordinarily consumed food.

For all these reasons, we have drawn three zones around the sites, with radii of 5 km, 20 km and 50 km respectively, for grouping the BASr baselines—a method inspired by the recent work of Snoeck et al. [104]. The fact that the sites are located in a very homogeneous, flat area (even if crossed by different hydrological basins), and the absence of geographical barriers (except rivers) to human mobility, make us more confident in applying the same dimensional criteria to different contexts.

The ‘5 km’ radius is here considered as the ‘site catchment area’ over which we assume the community exercised direct control and land exploitation. The ‘5–20 km’ radius is regarded as the ‘immediate hinterland’, reachable by foot in less than one day of travel. The ‘20–50 km’ radius is the ‘broader hinterland’, accessible only after more than one day of travel [112].

All the results of the strontium analysis in the following paragraphs have been compared to the baselines of these three distance categories.

## Results

The results of the analyses are shown in Table 1 and are discussed site by site in the following paragraphs.

**Table 1:** List of analysed burials, with archaeological, osteological and isotopic data (*Phase*: MBA = Middle Bronze Age; RBA = Recent Bronze Age. *Rite*: I = inhumation; C = cremation. *Sex*: M = male; F = female; I = indeterminate sex. *Age class*: INF1 = 0–6 yo; INF2 = 7–12 yo; JUV = 13–20 yo; AD = 21–40 yo; MAT = >40 yo. Sampled bone/tooth: M2 = second molar; M3 third molar).

Site	Grave n.	Burial group	Phase	Rite	Sex	Age Class	Age	Sampled bone/tooth	^87^Sr/^86^Sr	2SE	δ^18^O_VSMOW_	SD	δ^13^C_VPDB_	SD	Grave goods	Orientation of the body	Position of the body
Sant’Eurosia	1B	south of the tumuli	NA	I	M	AD	~30	M2 inf	0.709223	0.000011	23.39	0.05	-12.45	0.04		S-N	prone
Sant’Eurosia	2A	south of the tumuli	NA	I	F	JUV	~15	M2 inf	0.709101	0.000008	23.53	0.07	-13.19	0.02	shell in the mouth	N-S	supine
Sant’Eurosia	2B	south of the tumuli	NA	I	I	INF1	5–6	M2 sup	0.708854	0.000009	23.7	0.05	-13.46	0.04		S-N	supine
Sant’Eurosia	3	south of the tumuli	NA	I	I	INF2	~9	P4 sup	0.708953	0.000008	23.76	0.04	-13.69	0.04	bronze dagger	S-N	left side
Sant’Eurosia	6	south of the tumuli	NA	I	M	MAT	50+	M2 inf	0.708947	0.000009	23.49	0.05	-13.5	0.05	bronze dagger	S-N	prone
Sant’Eurosia	7	tumulus H	1	I	M	MAT	40–45	M2 sup	0.709262	0.000011	23.49	0.05	-13.98	0.06		SE-NW	left side
Sant’Eurosia	7	tumulus H	1	I	M	MAT	40–45	M2 sup	0.709329	0.000007	23.49	0.05	-13.98	0.06		SE-NW	left side
Sant’Eurosia	11	tumulus A	1	I	F	AD	20–30?	M2 sup	0.708438	0.000009	24.56	0.09	-14	0.05		W-E	supine
Sant’Eurosia	12	tumulus F	2	I	M	MAT	40–50	M2 inf	0.709154	0.000009	24.53	0.06	-14.09	0.06		S-N	supine
Sant’Eurosia	13	tumulus C	2	I	M	MAT	40–50	M2 inf	0.708625	0.000010	24.37	0.05	-13.12	0.03		SE-NW	supine
Sant’Eurosia	14A	tumulus D	2	I	F	AD	20–30	M2 sup	0.708686	0.000011	24.31	0.07	-14.28	0.04	earring	NE-SW	right side
Sant’Eurosia	16	tumulus D	2	I	M	AD	~30	M2 inf	0.708473	0.000008	23.66	0.08	-13.87	0.04		SE-NW	supine
Sant’Eurosia	17	tumulus B	2	I	I	INF2	8–9	M2 inf	0.708919	0.000010	23.16	0.05	-14.08	0.03		W-E	right side
Sant’Eurosia	19	between the tumuli	NA	I	I	INF2	~6	M2 sup	0.708984	0.000014	24.98	0.07	-14.3	0.04	bronze necklace, 3 shells in the mouth	S-N	left side
Sant’Eurosia	20	south of the tumuli	1	I	M	AD	?	P4 sup	0.709062	0.000008	24.71	0.08	-13.13	0.04		SW-NE	NA
Sant’Eurosia	23	tumulus A	1	I	M	AD	30–40	M2 sup	0.709210	0.000010	24.51	0.09	-13.97	0.04		SW-NE	left side
Sant’Eurosia	24	tumulus A	1	I	M	AD	30–35	M2 sup	0.709381	0.000008	24.23	0.06	-13.91	0.01		E-W	left side
Sant’Eurosia	25	tumulus A	1	I	M	AD	?	M2 inf	0.708875	0.000012	24.35	0.07	-13.46	0.04	steatie beads	SW-NE	left side
Sant’Eurosia	27	tumulus D	2	I	F	AD	20–25	M2 sup	0.709027	0.000010	24.26	0.08	-14.12	0.05	awl	W-E	right side
Sant’Eurosia	28	tumulus A	1	I	M	MAT	40–50	M2 sup	0.708660	0.000009	24.31	0.05	-14.28	0.03	pot (robbed grave)	SW-NE	left side
Sant’Eurosia	US424	tumulus B	NA	I	M	AD	30–40	M2 sup	0.708805	0.000009	24.01	0.08	-13.31	0.05			
Casinalbo	4	A	RBA2	C	F	AD	NA	petrous portion	0.709138	0.000010					fibula, earring		
Casinalbo	15	A	RBA1	C	M	AD	NA	petrous portion	0.708741	0.000008							
Casinalbo	16	A	RBA2	C	I	INF1	NA	petrous portion	0.708650	0.000007							
Casinalbo	40	Y	RBA1	C	F	AD	NA	petrous portion	0.708598	0.000008					bronze ornament frag., earrings, glass beads, antler wheel-shaped pinhead		
Casinalbo	41	Y	RBA1	C	M	AD	NA	petrous portion	0.708714	0.000009							
Casinalbo	131	I	RBA1	C	F	JUV	NA	petrous portion	0.708619	0.000010							
Casinalbo	149	I	MBA2B+3A	C	F	MAT	NA	petrous portion	0.709206	0.000009					bronze ornament frag.		
Casinalbo	163	K	RBA2	C	F	MAT	NA	petrous portion	0.708569	0.000010							
Casinalbo	184	K	MBA3B	C	I	INF1	NA	petrous portion	0.708547	0.000009							
Casinalbo	185	K	RBA1	C	M	AD	NA	petrous portion	0.708706	0.000008					sword/dagger frag.		
Casinalbo	189	K	RBA1	C	F	AD	NA	petrous portion	0.709002	0.000011							
Casinalbo	192	I	MBA3B	C	F	AD	NA	petrous portion	0.709120	0.000008					bronze ornament frag.		
Casinalbo	230	K	RBA1	C	F	AD	NA	petrous portion	0.708960	0.000010							
Casinalbo	242	K	RBA2	C	F	JUV	NA	petrous portion	0.708501	0.000010							
Casinalbo	264	En	MBA2B+3A	C	M	MAT	NA	petrous portion	0.709253	0.000011							
Casinalbo	274	E	RBA2	C	M	AD	NA	petrous portion	0.708675	0.000010							
Casinalbo	289	E	MBA2B+3A	C	M	AD	NA	petrous portion	0.708624	0.000008							
Casinalbo	305	E	MBA3B	C	F	AD	NA	petrous portion	0.708742	0.000011							
Casinalbo	333	D	RBA	C	F	AD	NA	petrous portion	0.709136	0.000007					bronze pin		
Casinalbo	357	D	RBA	C	I	INF1	NA	petrous portion	0.709046	0.000009							
Casinalbo	474	E	MBA2B+3A	C	M	AD	NA	petrous portion	0.708618	0.000009							
Casinalbo	488	C	MBA2B+3A	C	M	MAT	NA	petrous portion	0.708752	0.000012							
Casinalbo	491-petrous	C	MBA2B+3A	C	I	INF1	NA	petrous portion	0.708909	0.000011							
Casinalbo	491-M2	C	MBA2B+3A	C	I	INF1	NA	M2 sup	0.708959	0.000009							
Casinalbo	499	C	MBA2B+3A	C	M	AD	NA	petrous portion	0.709686	0.000010							
Scalvinetto/Fondo Paviani	12	NA	NA	C	I	JUV		petrous portion	0.709928	0.000010					NA		
Scalvinetto/Fondo Paviani	15	NA	NA	C	M	AD		petrous portion	0.709650	0.000012					NA		
Scalvinetto/Fondo Paviani	27	NA	NA	C	M	AD		petrous portion	0.709696	0.000010					NA		
Scalvinetto/Fondo Paviani	30	NA	NA	C	M	MAT		petrous portion	0.709451	0.000009					NA		
Scalvinetto/Fondo Paviani	49	NA	NA	C	F	AD		petrous portion	0.710137	0.000012					NA		
Scalvinetto/Fondo Paviani	51	NA	NA	C	F	AD		petrous portion	0.710183	0.000009					NA		
Scalvinetto/Fondo Paviani	52	NA	NA	C	I	INF 1		petrous portion	0.709899	0.000010					NA		
Scalvinetto/Fondo Paviani	58	NA	NA	C	I	INF 1		M2 sup	0.709863	0.000010					NA		
Scalvinetto/Fondo Paviani	61	NA	NA	C	M	AD		petrous portion	0.709944	0.000012					NA		
Scalvinetto/Fondo Paviani	72	NA	NA	C	M	AD		petrous portion	0.709199	0.000010					NA		
Scalvinetto/Fondo Paviani	74	NA	NA	C	F	MAT		petrous portion	0.710256	0.000010					NA		
Scalvinetto/Fondo Paviani	91	NA	NA	C	M	AD		petrous portion	0.709439	0.000011					NA		
Scalvinetto/Fondo Paviani	96	NA	NA	C	F	AD		petrous portion	0.709845	0.000012					NA		
Scalvinetto/Fondo Paviani	102	NA	NA	C	F	AD		petrous portion	0.709532	0.000012					NA		
Scalvinetto/Fondo Paviani	116	NA	NA	C	M	MAT		petrous portion	0.709490	0.000010					NA		
Scalvinetto/Fondo Paviani	156	NA	NA	C	M	AD		petrous portion	0.710099	0.000013					NA		
Scalvinetto/Fondo Paviani	172	NA	NA	C	F	JUV		petrous portion	0.710195	0.000013					NA		
Scalvinetto/Fondo Paviani	174	NA	NA	C	I	JUV		petrous portion	0.709593	0.000011					NA		
Scalvinetto/Fondo Paviani	176	NA	NA	C	I	INF 2		petrous portion	0.709952	0.000011					NA		
Scalvinetto/Fondo Paviani	178	NA	NA	C	F	AD		petrous portion	0.710086	0.000010					NA		
Scalvinetto/Fondo Paviani	191	NA	NA	C	F	AD		petrous portion	0.710233	0.000009					NA		
Scalvinetto/Fondo Paviani	196	NA	NA	I	M	JUV	12–18	M2 sup	0.709144	0.000005	24.16	0.06	-8.15	0.04	NA	E-W	supine
Scalvinetto/Fondo Paviani	209	NA	NA	I	I	INF 2	5–9	M2 inf	0.709892	0.000008	24.79	0.09	-4.18	0.04	NA	E-W	supine
Scalvinetto/Fondo Paviani	225	NA	NA	C	M	AD		petrous portion	0.709235	0.000013					NA		
Scalvinetto/Fondo Paviani	231	NA	NA	I	I	JUV	10–14	M2 inf	0.709792	0.000008	25.01	0.08	-12.56	0.03	NA	E-W	supine
Scalvinetto/Fondo Paviani	236	NA	NA	I	M	AD	?	M2 inf	0.710150	0.000009	25.46	0.08	-4.48	0.03	NA	E-W	supine
Scalvinetto/Fondo Paviani	244	NA	NA	I	F	AD	?	M2 inf	0.708712	0.000008	26.06	0.07	-13.35	0.06	NA	E-W	supine
Scalvinetto/Fondo Paviani	246-M2	NA	NA	I	F	AD-MAT	?	M2 sup	0.719605	0.000009	26.9	0.07	-14.02	0.03	NA	S-N	supine
Scalvinetto/Fondo Paviani	246-M3	NA	NA	I	F	AD-MAT	?	M3 sup	0.717075	0.000009	26.25	0.08	-11.68	0.02	NA	S-N	supine
Scalvinetto/Fondo Paviani	261	NA	NA	C	F	MAT		petrous portion	0.710104	0.000009					NA		
Scalvinetto/Fondo Paviani	271	NA	NA	C	I	INF 2		petrous portion	0.709746	0.000008					NA		
Scalvinetto/Fondo Paviani	274	NA	NA	I	F	AD	35–45	M2 sup	0.708484	0.000010	24.08	0.07	-10.19	0.04	NA	E-W	supine
Scalvinetto/Fondo Paviani	278	NA	NA	I	F	MAT	25–35	M2 sup	0.710407	0.000007	26.42	0.08	-11.86	0.06	NA	S-N	supine
Scalvinetto/Fondo Paviani	285	NA	NA	I	F	AD	30–40	M2 sup	0.709620	0.000007	26.67	0.04	-13.78	0.03	NA	E-W	supine
Scalvinetto/Fondo Paviani	288	NA	NA	I	F	JUV-AD	?	M2 sup	0.710189	0.000006	25.4	0.08	-12.59	0.02	NA	E-W	supine
Scalvinetto/Fondo Paviani	299	NA	NA	I	F	JUV-AD	?	M2 inf	0.709637	0.000009	26.56	0.09	-11.42	0.04	NA	N-S	supine
Scalvinetto/Fondo Paviani	310	NA	NA	I	F	AD	?	M2 inf	0.709471	0.000008	24.43	0.06	-5.44	0.03	NA	E-W	supine
Scalvinetto/Fondo Paviani	313-M2	NA	NA	I	F	AD	25–30	M2 sup	0.708094	0.000007	25.37	0.09	-12.16	0.022	NA	E-W	supine
Scalvinetto/Fondo Paviani	313-M3	NA	NA	I	F	AD	25–30	M3 sup	0.709568	0.000013	25.41	0.06	-3.08	0.03	NA	E-W	supine
Scalvinetto/Fondo Paviani	315	NA	NA	I	F	AD	25–30	M2 sup	0.712438	0.000007	26.95	0.05	-13.36	0.03	NA	N-S	supine
Scalvinetto/Fondo Paviani	325	NA	NA	I	F	AD	25–30	M2 inf	0.712178	0.000006	27.63	0.06	-13.45	0.04	NA	E-W	supine
Scalvinetto/Fondo Paviani	326	NA	NA	I	F	MAT	40–50	M2 sup	0.709352	0.000008	25.48	0.07	-13.06	0.03	NA	E-W	supine
Scalvinetto/Fondo Paviani	327	NA	NA	I	F	AD	25–40	M2 sup	0.709413	0.000007	26.93	0.04	-13.82	0.05	NA	E-W	supine
Scalvinetto/Fondo Paviani	327	NA	NA	I	F	AD	25–40	M2 sup	0.709279	0.000007	-	-	-		NA	E-W	supine
Scalvinetto/Fondo Paviani	328	NA	NA	I	M	JUV	18–20	M2 inf	0.709253	0.000010	26.30	0.07	-11.96	0.04	NA	E-W	supine
Scalvinetto/Fondo Paviani	329	NA	NA	I	F	AD	?	M2 sup	0.709093	0.000008	25.7	0.09	-11.86	0.07	NA	E-W	supine
Scalvinetto/Fondo Paviani	344	NA	NA	I	F	AD-MAT	?	M2 sup	0.708009	0.000010	26.48	0.06	-12.29	0.03	NA	E-W	lateral left
Scalvinetto/Fondo Paviani	345	NA	NA	I	M	AD	20–30	M2 inf	0.709021	0.000008	24.77	0.06	-8.01	0.04	NA	E-W	supine
Scalvinetto/Fondo Paviani	352	NA	NA	I	I	JUV	12–15	M2 inf	0.709252	0.000008	25.02	0.05	-4.97	0.04	NA	E-W	supine
Scalvinetto/Fondo Paviani	373	NA	NA	I	F	AD	20–35	M2 sup	0.708992	0.000012	25.47	0.09	-5.19	0.03	NA	S-N	supine
Scalvinetto/Fondo Paviani	398	NA	NA	I	M	AD	~25	M2 inf	0.710022	0.000008	26.24	0.06	-12.25	0.04	NA	E-W	supine
Scalvinetto/Fondo Paviani	400	NA	NA	I	F	AD	35–40	M2 sup	0.709027	0.000010	26.13	0.07	-6	0.04	NA	E-W	supine
Scalvinetto/Fondo Paviani	401	NA	NA	I	M	AD	35–40	M2 sup	0.710279	0.000005	25.18	0.09	-4.12	0.04	NA	E-W	supine
Scalvinetto/Fondo Paviani	403	NA	NA	I	M	AD	?	M2 sup	0.709226	0.000010	26.13	0.06	-3.46	0.03	NA	E-W	supine
Scalvinetto/Fondo Paviani	404	NA	NA	I	F	AD	20–30	M2 sup	0.710136	0.000006	26.08	0.08	-5.93	0.03	NA	E-W	supine
Scalvinetto/Fondo Paviani	405-M2	NA	NA	I	M	AD	30–40	M2 sup	0.719292	0.000010	24.77	0.05	-6.3	0.04	NA	E-W	supine
Scalvinetto/Fondo Paviani	405-M3	NA	NA	I	M	AD	30–40	M3 sup	0.711976	0.000014	25.75	0.06	-3.49	0.06	NA	E-W	supine
Scalvinetto/Fondo Paviani	424	NA	NA	C	F	AD		petrous portion	0.709874	0.000009					NA		
Scalvinetto/Fondo Paviani	433	NA	NA	C	I	AD		petrous portion	0.709858	0.000009					NA		
Scalvinetto/Fondo Paviani	454	NA	NA	C	M	AD		petrous portion	0.710098	0.000014					NA		
Scalvinetto/Fondo Paviani	456	NA	NA	C	F	AD		petrous portion	0.709953	0.000013					NA		
Scalvinetto/Fondo Paviani	474	NA	NA	C	M	MAT		petrous portion	0.710156	0.000018					NA		
Scalvinetto/Fondo Paviani	485	NA	NA	C	M	AD		petrous portion	0.709704	0.000012					NA		
Scalvinetto/Fondo Paviani	527	NA	NA	I	I	JUV	12–18	M2 sup	0.709987	0.000006	26.22	0.07	-6.84	0.03	NA	NE-SW	supine
Scalvinetto/Fondo Paviani	Fauna (416)	NA	NA	-	-	-	-	tooth enamel	0.710128	0.000006	23.78	0.07	-2.78	0.04	-	-	-

### Sant’Eurosia

**Fig 3:**
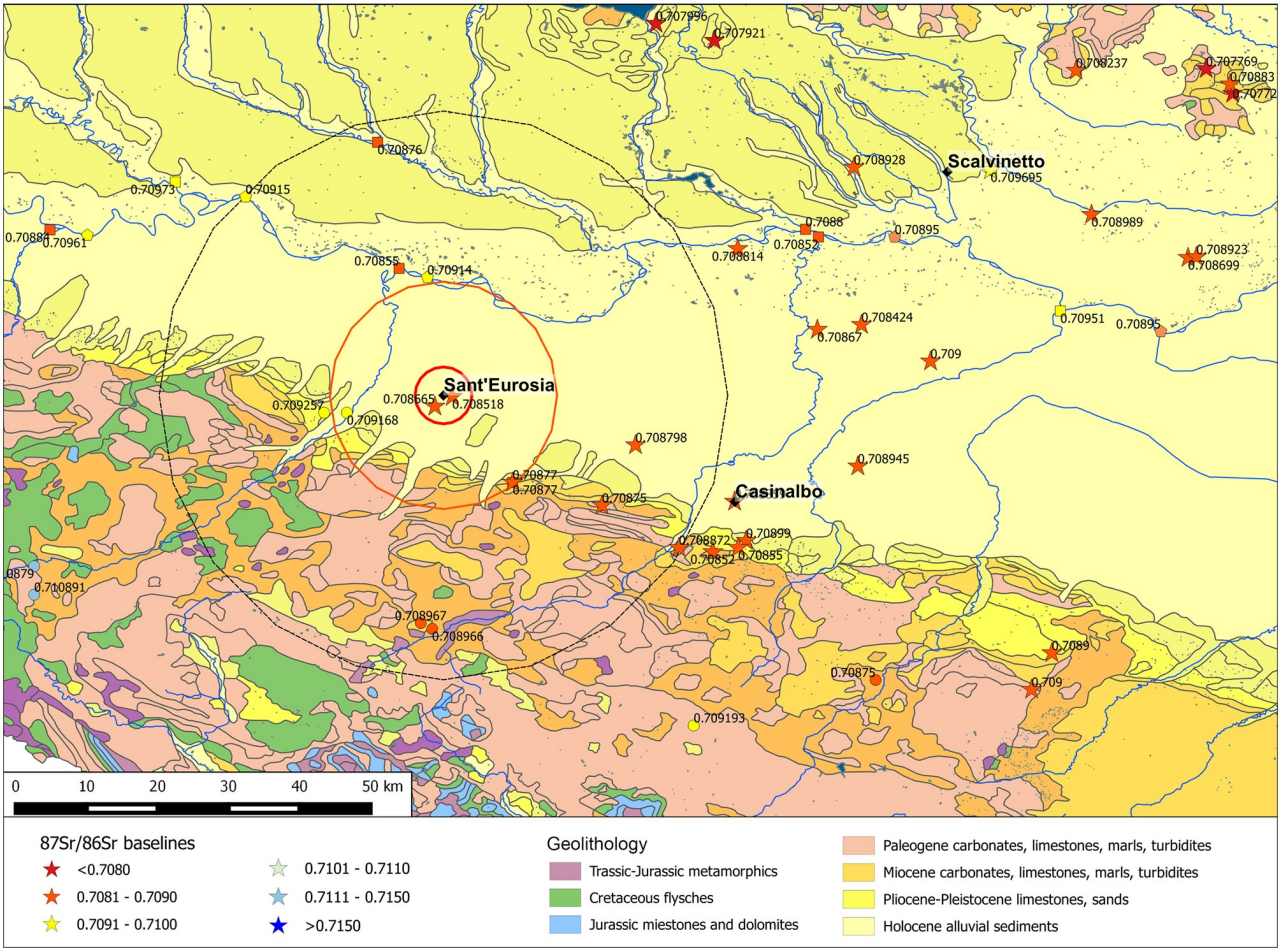
Sant’Eurosia site with the buffer zones and the available BASr baselines. Stars represent the baselines obtained from vegetal/faunal samples, circles on mineral waters, pentagons on Po river waters, squares on tributary river waters. The map is constructed by using public domain wms data downloadable from http://wms.pcn.minambiente.it/ogc?map=/ms_ogc/WMS_v1.3/Vettoriali/Carta_geolitologica.map under a CC BY license, with permission from Geoportale Nazionale and plotting data from S1 Table.

Strontium, oxygen and carbon isotopes were analysed in 20 tooth enamel samples from 20 individuals. Strontium baselines for Sant’Eurosia’s 5 km territory range between 0.7082 and 0.7091 (Fig 3). The variability of the 5 km ^87^Sr/^86^Sr baselines is significantly expanded by the values of the animal samples recovered from the cemetery. Considering that the only available faunal remains were of *sus scrofa*, we cannot be absolutely certain that the animals grew up and consumed food in the vicinity of the site. We cannot rule out that pigs might have been transported from other sites, in exchange for goods or as gifts.

**Fig 4:**
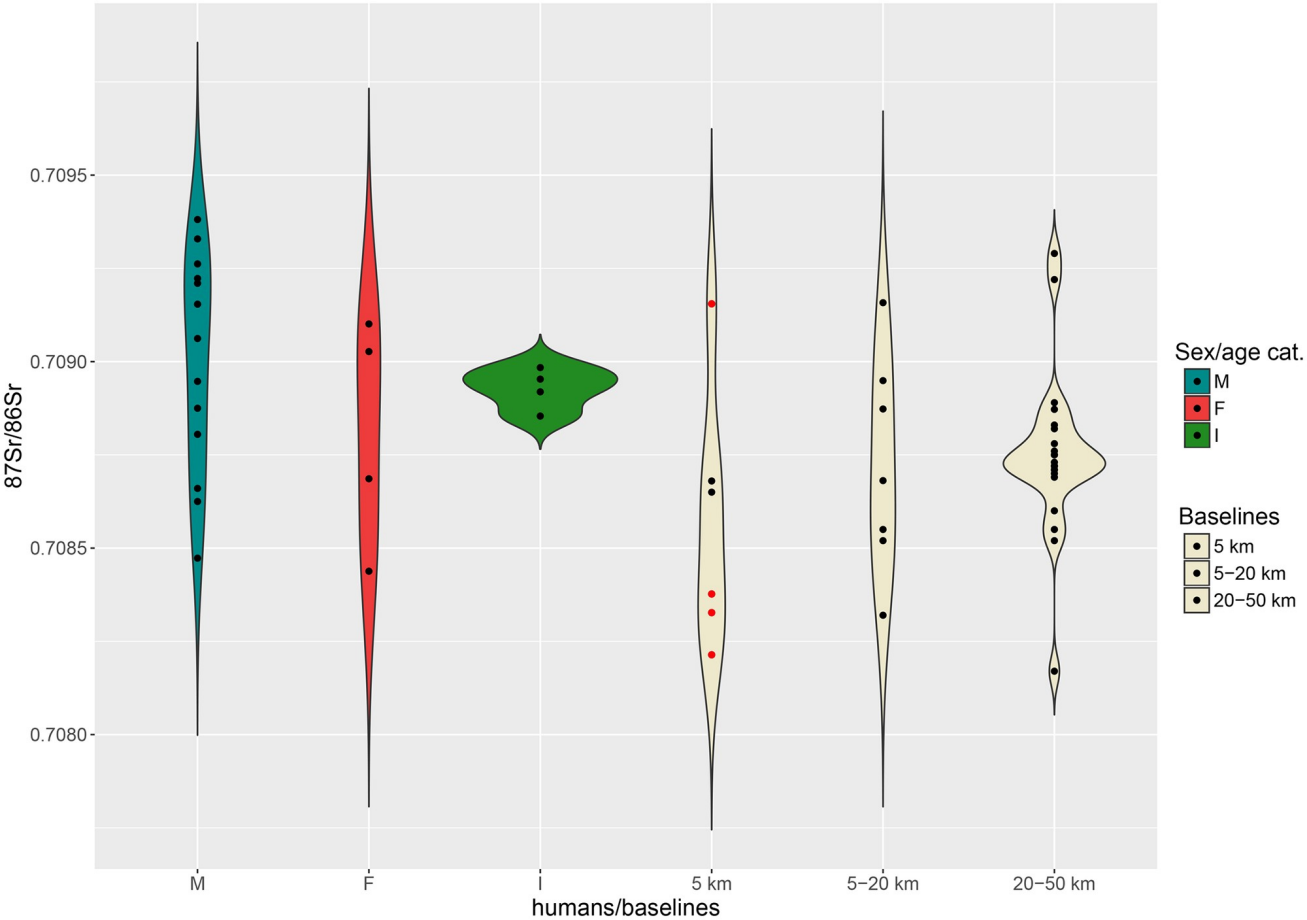
Violin plot. Distribution of the ^87^Sr/^86^Sr values from adult males (M), females (F), 5–9 years old individuals (I) and 5 km, 5–20 km, 20–50 km baselines at Sant’Eurosia. Red dots among baselines represent archaeological fauna samples.

By contrast, ^87^Sr/^86^Sr values measured on two grape juices from Parma are close to 0.7087, a ratio similar, although slightly lower, to that of the four children (*Infans 1*) analysed from the cemetery (0.7089–0.7090). The baselines of the immediate hinterland (5–20 km) range between 0.7083 and 0.7092, and between 0.7082 and 0.7093 for the broader hinterland (Fig 4).

In spite of the smaller number of female individuals, male and female distributions do not differ considerably and are consistent with the 5 and 5–20 km baselines (Fig 4). Five adult males may come from the broader hinterland and two even from an even greater distance (values greater than 0.7093).

The adult individuals do not seem to be isotopically concentrated but rather appear varied, likely as a consequence of a high degree of human mobility within the hinterland.

No correlation has been found between isotope ratios and orientiation/position of the body in the graves.

**Fig 5:**
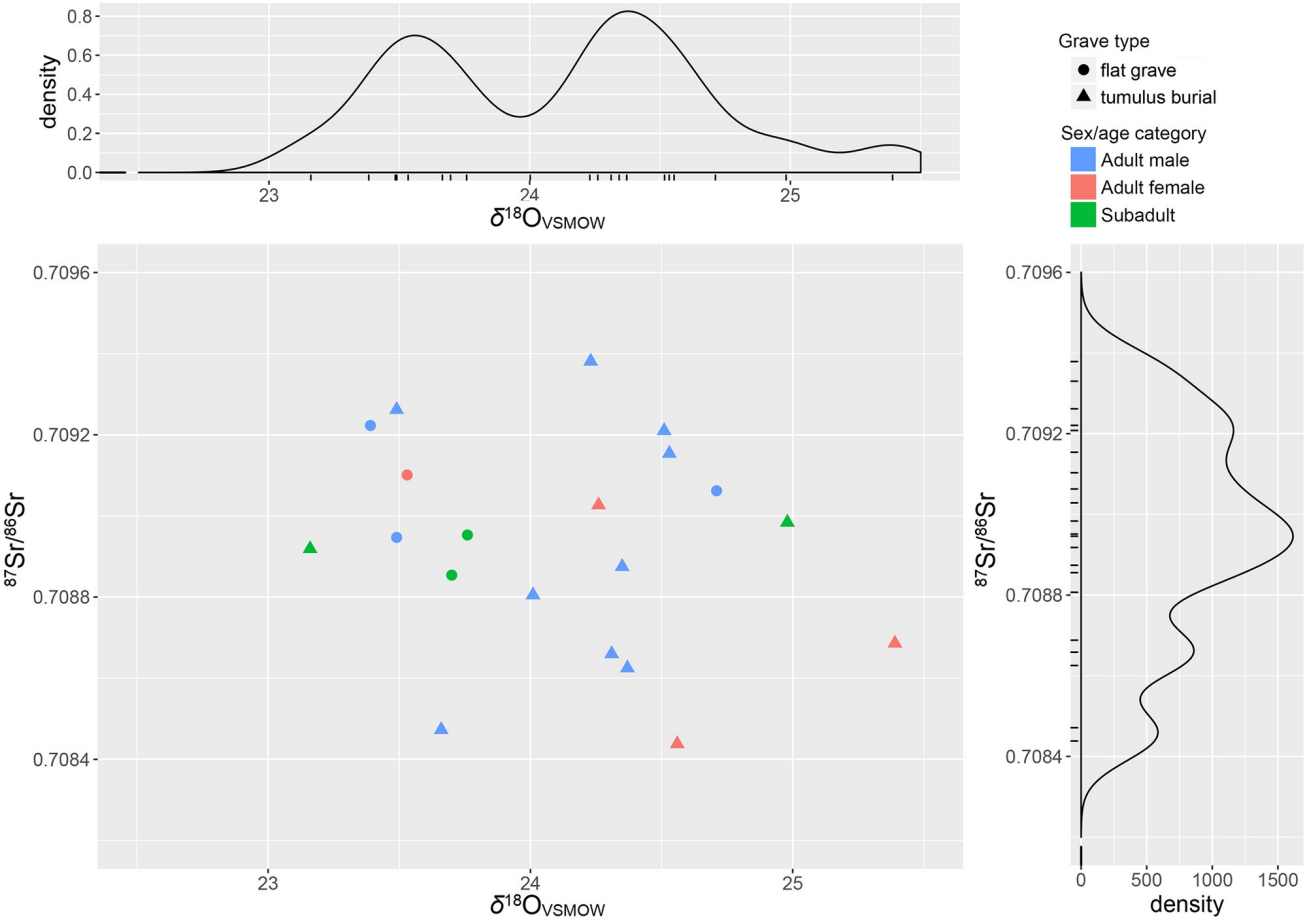
Sant’Eurosia scatter and density plots of ^87^Sr/^86^Sr and δ^18^O_VSMOW (carbonate)_. Symbol shape and colours indicate the grave type and the sex/age category of the individuals, respectively.

δ^18^O_carb_ values from carbonates (VSMOW) in human tooth enamel vary between 23.16‰ and 25.39‰ for a total range of 2.23‰ (Fig 5). The distributions of ratios among the three sex/age categories appear quite similar. Two groups are clearly distinguishable in the density plot, one above and the other below 24.01‰, which seem to be partially correlated with the type of grave and the presence of bronze grave goods. Except for one case (t. 20), all the flat graves south of the tumuli show very similar strontium and oxygen isotope ratios, along with some of the tumulus burials. Also, the two burials including bronze daggers are characterized by δ^18^O values lower than 24.01‰ (TT. 3 and 6). However, a Mann–Whitney U showed no significant difference between the distributions of δ^18^O values in flat graves and those under tumuli (p = 0.11).

Converted to drinking water values, the δ^18^O_VSMOW-dw_ range for Sant’Eurosia individuals is between -11.8‰ and -8.3‰, the mean is −10.3‰± 1.5 and standard deviation is 0.93‰. These values are slightly lower than the local meteoric δ^18^O, reported in the literature, of about 1–1.5‰, possibly as consequence of slightly different climatic conditions. Modern rainfall, river waters and ground water in the Parma province range respectively between -10.2‰ and -7.3‰, between -9.5‰ and -8.0‰, and between -8.5‰ and -8.1‰ [49,50,113,114].

δ^13^C_VPDB_ values measured on the same apatite samples range between -14.30‰ and -12.45‰. According to Kellner and Schoeninger [115], these values are consistent with a C_3_ protein/energy based diet, possibly with slight supplement of C_4_ (or, unlikely given the site location, of marine food) for those individuals with the highest values. The Sant’Eurosia enamel δ^13^C seem to reflect the range of approximately -21‰ ‒ -19‰ on the collagen.

**Fig 6:**
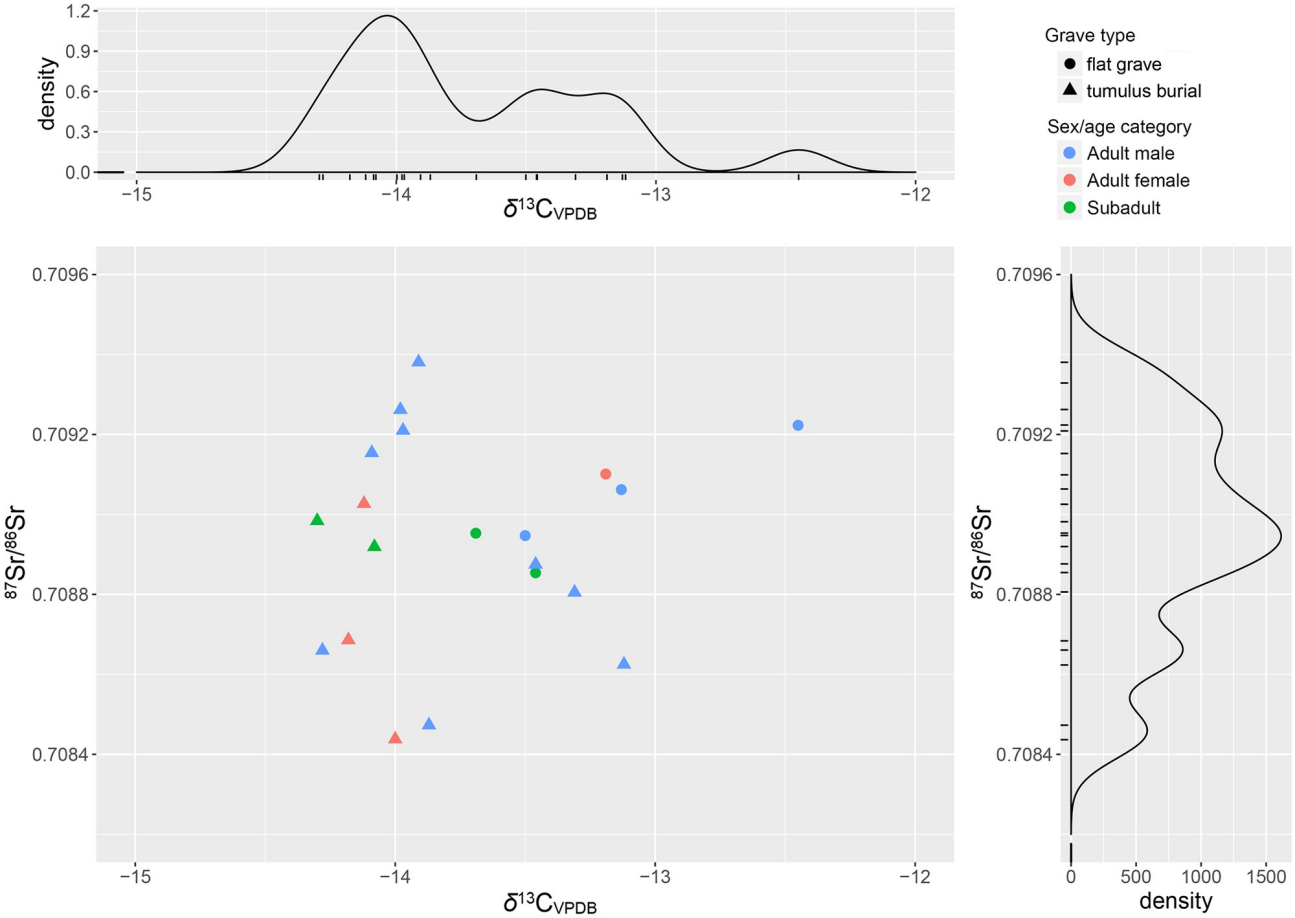
Sant’Eurosia scatter and density plots of ^87^Sr/^86^Sr and δ^13^C_VPDB_. Symbol shape and colors indicate the grave type and the sex/age category of the individuals, respectively.

Again, the density plot shows two clearly distinguishable groups (Fig 6), one below the threshold of -13.87‰ exclusively including graves under tumuli, and another above -13.69‰ including both flat graves and inhumations under tumuli. The difference in δ^13^C between the two groups of burials was signifcant in a Mann–Whitney U test (p = 0.01).

Moreover, as with the oxygen isotope results, the two burials with bronze daggers have a similar δ^13^C value (T. 3 = -13.69‰; T. 6 = -13.50‰). The correlation coefficient between oxygen and carbon δ-values is R = -0.38 [95% CI: -0.70–0.07], indicating no strong relationship.

The fact that both oxygen and carbon isotope ratios seem at least partially correlated with the type of grave might support the idea that the community buried at Sant’Eurosia includes groups with different mobility/dietary patterns, and emphasize their distinct social membership through funerary custom and the segregation of their dead in a specific part of the cemetery. According to the four available ^14^C dates, the burials under the tumuli tend to be more ancient that those in the flat graves located in the southern area of the cemetery [92]. It might therefore be that the provenance of the individuals and/or dietary behaviour partially changed over time at Sant’Eurosia, along with the ritual customs and the burial sector. The picture yielded by the strontium/oxygen isotope analysis is that of a community mobile in the local territory across the generations, plausibly in the periodic search for virgin and exploitable soils. The community nontheless persisted to bury their dead in the same funerary area, clearly marked in the landscape by the monumental tumuli.

Paleoclimatic factors could also help to explain the variations. If flat graves are more recent that the burials under the tumuli, an increase of δ^13^C over the decades might have been caused by a reduction of mean annual precipitation [116,117]. Additional and more precise radiocarbon dates on Sant’Eurosia’s human bones are necessary to verify any relationship between changes in stable isotope composition, chronology and paleoclimatic changes.

### Casinalbo

The test of correspondence between M2 and petrous portion in t. 491 gave very similar results (petrous portion = 0.708909; M2 = 0.708959), demonstrating the reliability of strontium isotope analyses of cremated petrous portions.

**Fig 7:**
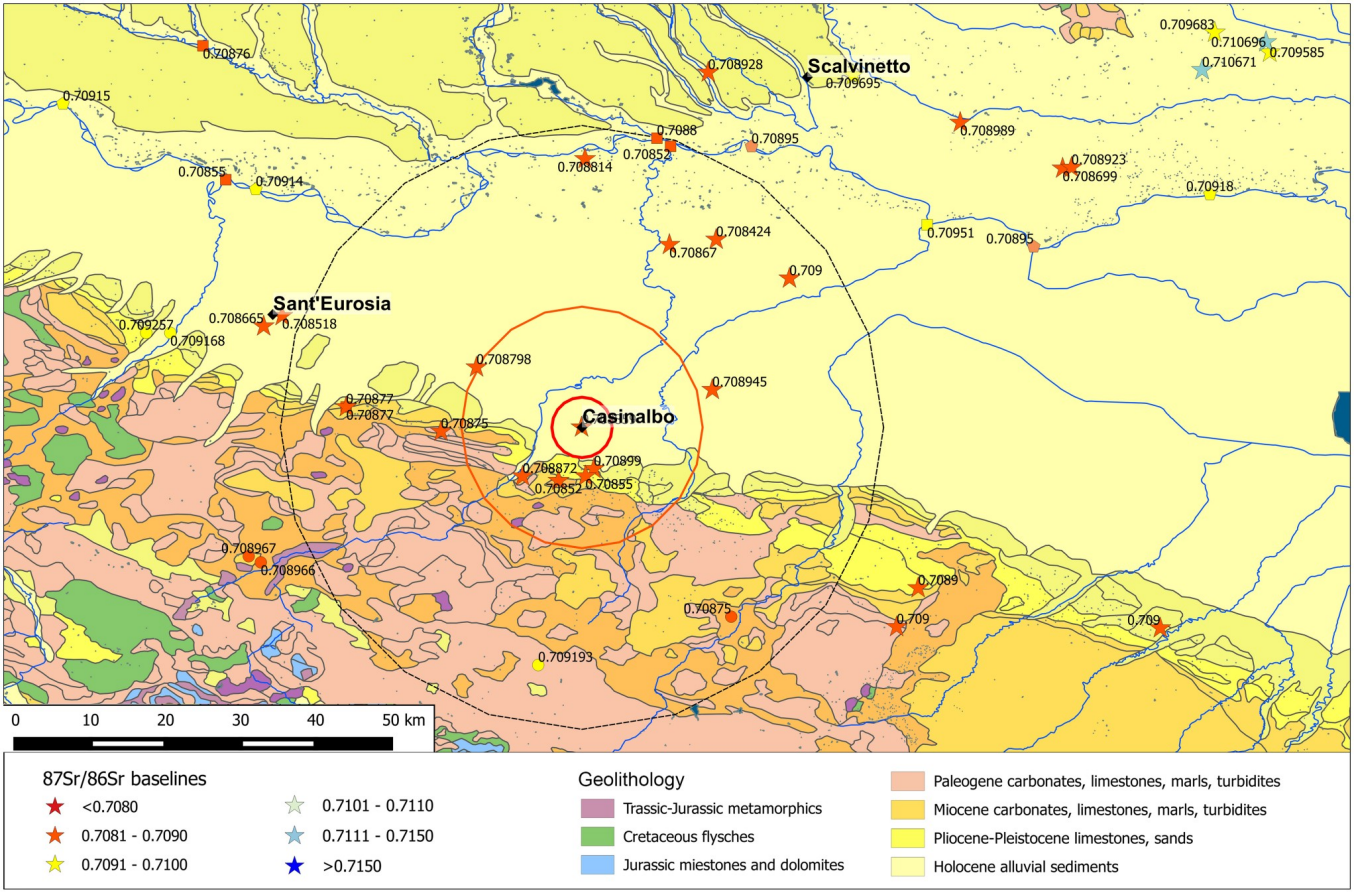
Casinalbo with the buffer zones and the available BASr baselines. Stars represent the baselines obtained from vegetal/faunal samples, circles on mineral waters, pentagons on Po river waters, squares on tributary river waters. The map is constructed by using public domain wms data downloadable from http://wms.pcn.minambiente.it/ogc?map=/ms_ogc/WMS_v1.3/Vettoriali/Carta_geolitologica.map under a CC BY license, with permission from Geoportale Nazionale and plotting data from S1 Table.

Strontium baselines for the territory surrounding Casinalbo range between 0.7085 and 0.7089 within a radius of 5 km from the site, between 0.7084 and 0.7090 in the immediate hinterland (5–20 km) and between 0.7084 and 0.7090 in the broader hinterland (20–50 km) (Fig 7). The overall geological homogeneity of the Emilian alluvial plain reflects the variability of the strontium baselines at different distances. On the one hand, such uniformity complicates the identification of the individuals’ provenance; on the other hand, it simplifies the identification of ‘outliers’, and increases the probability of their ‘foreign’ origin.

**Fig 8:**
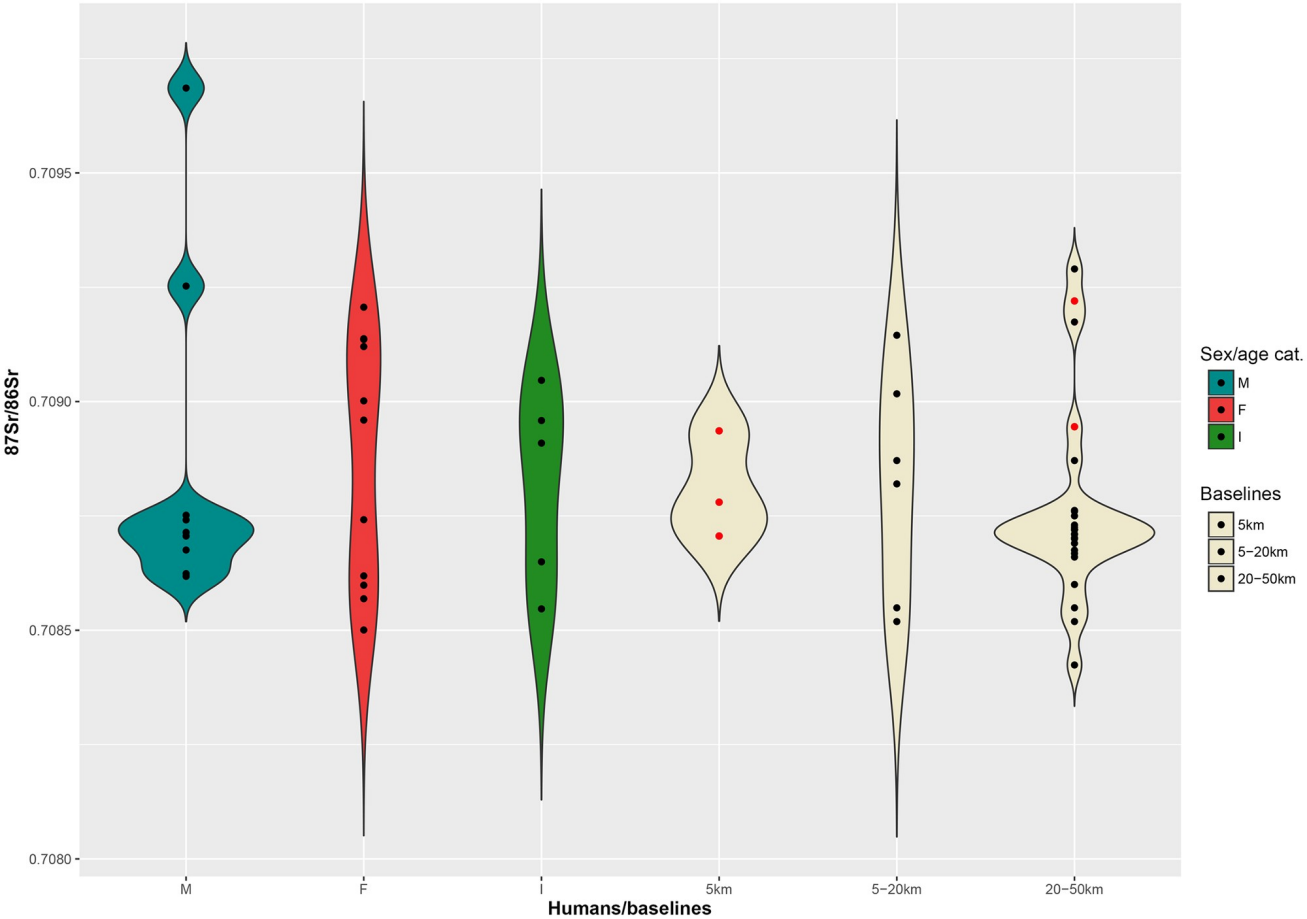
Violin plot. Distribution of the ^87^Sr/^86^Sr values from adult males (M), females (F), *Infans 1* (I) cremations and 5 km, 5–20 km, 20–50 km baselines at Casinalbo. Red dots among baselines represent archaeological fauna samples.

Strontium isotope ratios for the majority of adult males are instead much more concentrated in the range 0.7086–0.7088, although two male outliers, t. 264 and t. 499, show greater values (Fig 8). Their ratios, 0.7093 and 0.7097 respectively, might indicate their more distant origins, possibly between the lower Adige/lower Brenta River valleys, where local baselines range between 0.7089 and 0.7107.

Adult female values range from 0.7085 to 0.7092 and are consistent with the 5–20 km baseline distribution. Some of the females could also have been included in the Casinalbo burial community also as a consequence of marriage mobility within a radius of 20 km.

The distribution of strontium isotope ratios for the *Infans 1* age category (0–6 years of age) slightly exceeds the 5 km baseline range but is less broad than the 5–20 km range. Young children have had less opportunity to move, and therefore are expected to be consistent with local baselines. Moreover, young individuals could move either with or without other family members to join other communities in a neighbouring area. Thus, some of the children buried in the Casinalbo urnfield might have come from outside the site area, from a distance of up to 20 km, but it is impossible to establish if they moved from a place near to Casinalbo during their lifetime, or if they lived in the hinterland and were just buried there. In the latter case, less probable, the urnfield could include people not just from the *terramara* but also from other neighbouring sites.

Some further characteristics of mobility patterns can be suggested by integrating the archaeological data [12]. For example, burials from the earlier phases (1500–1350 BC) tend to show more variable strontium isotope ratios, while the later burials seem more concentrated around the 5 km or 5–20 km baselines. This pattern may be connected to the progressive stabilization of the settlement and its relationships with surrounding communities, as well as with more distant places. After a phase of absorbing people from different places, it could be that the community was able to grow internally, without the need or desire to assimilate further outsiders.

It is also intriguing to observe that adult females accompanied by grave goods (ornaments) are generally characterized by higher strontium isotope ratios (0.7091–0.7092), possibly indicating a more distant, but similar, provenance, with the single exception of t. 40 (0.7086); adult females without grave goods, by contrast, tend to have lower ratios, closer to the 5 km or 5–20 km baselines (0.7085–0.7090). This evidence might be interpreted in terms of local and non-local females having distinct cultural customs and statuses, expressed through the burial ritual and furnishings.

### Scalvinetto inhumations

**Fig 9:**
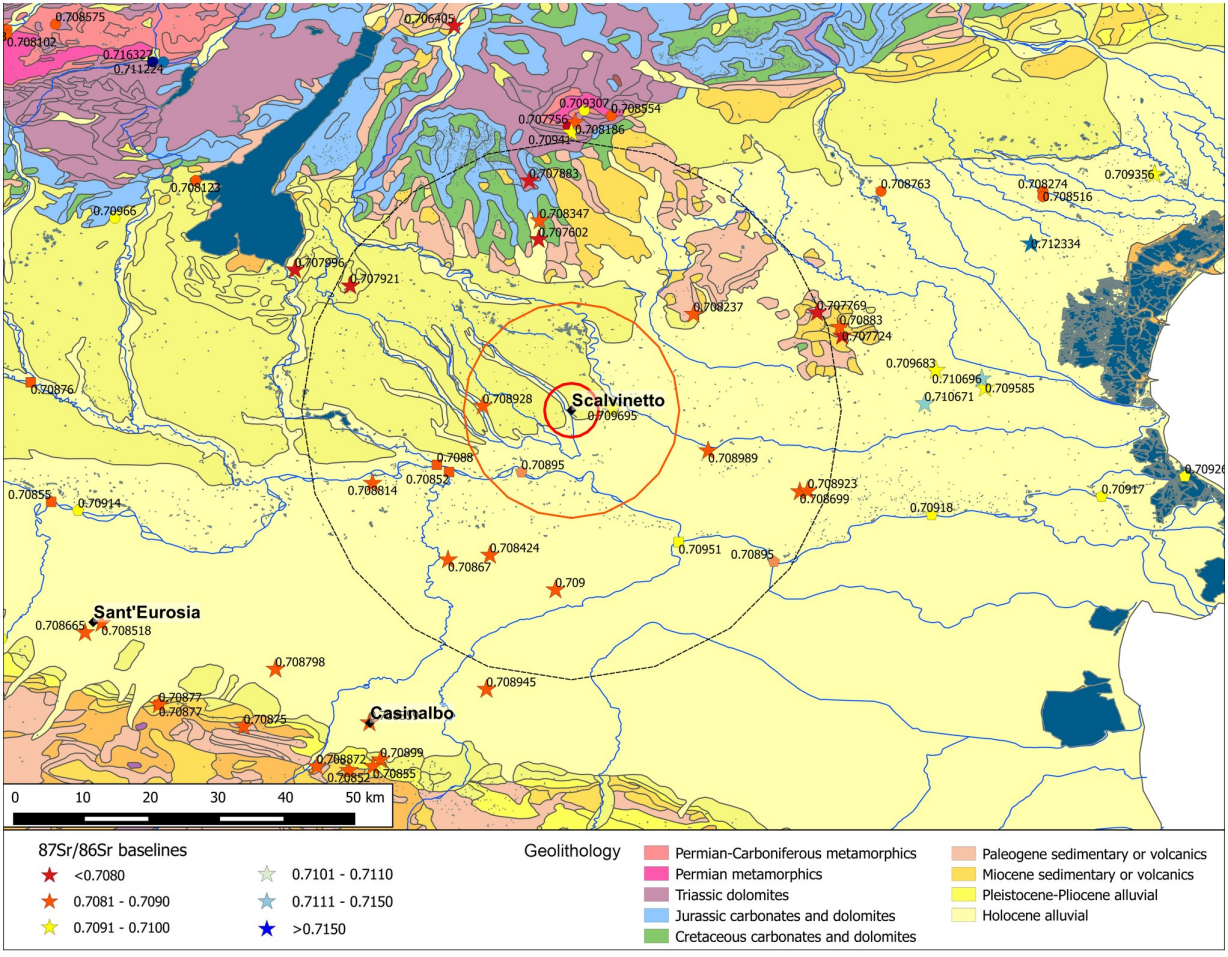
Scalvinetto/Fondo Paviani with the buffer zones and the available BASr baselines. Stars represent the baselines obtained from vegetal/faunal samples, circles on mineral waters, pentagons on Po river waters, squares on tributary river waters. The map is constructed by using public domain wms data downloadable from http://wms.pcn.minambiente.it/ogc?map=/ms_ogc/WMS_v1.3/Vettoriali/Carta_geolitologica.map under a CC BY license, with permission from Geoportale Nazionale and plotting data from S1 Table.

The 30 analysed individuals show a wide distribution of ^87^Sr/^86^Sr values, about four times greater than the variability at Casinalbo and Sant’Eurosia. This is partially due to the amplitude of the isotope ratio variation north of the River Po, specifically, in the Verona Province, characterized by very different geolithological zones even in a relatively restricted territory (Fig 9), but also due to the high degree of permeability of the Scalvinetto/Fondo Paviani community to non-local individuals.

**Fig 10:**
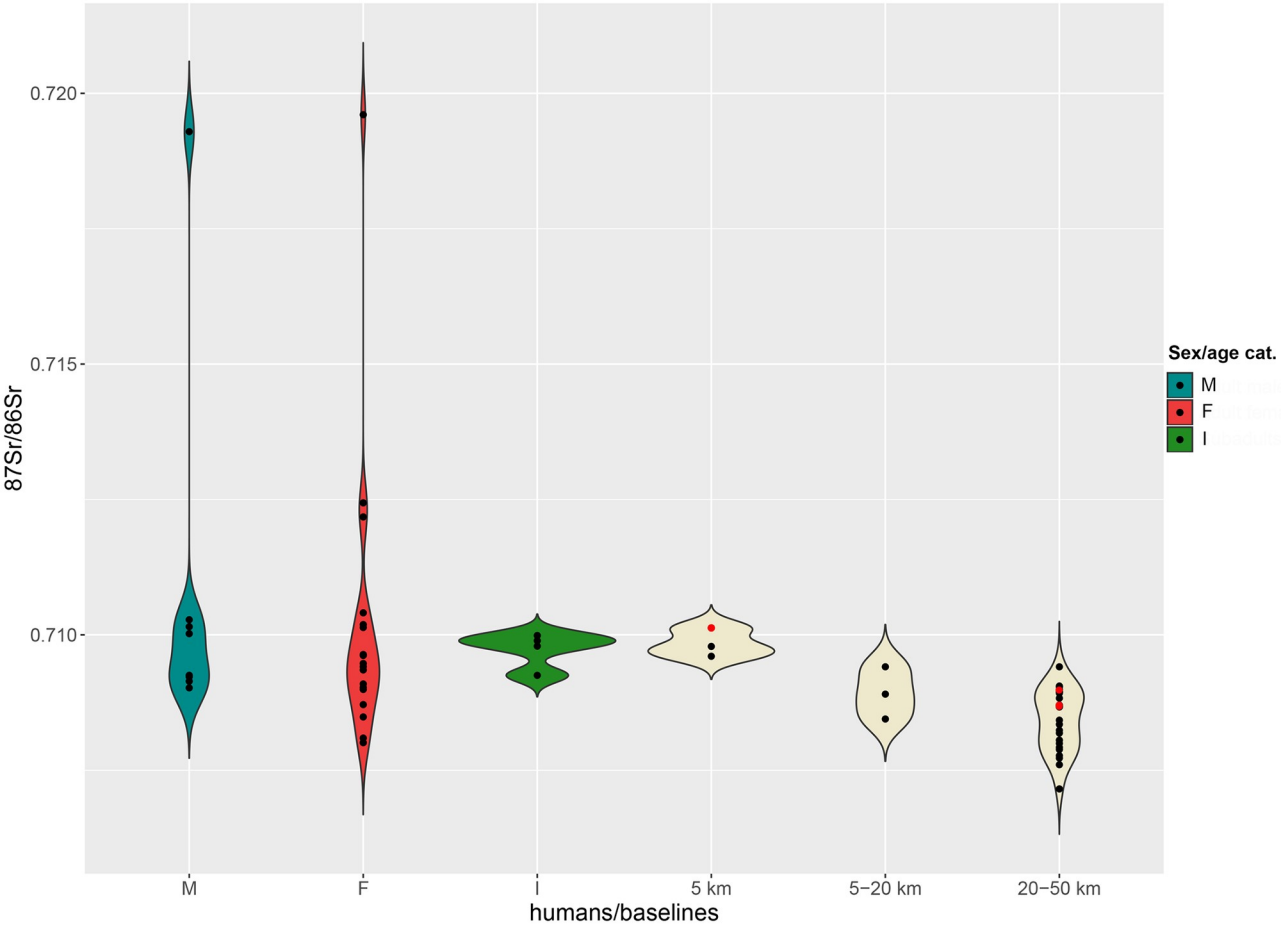
Violin plot. Distribution of the ^87^Sr/^86^Sr values from adult male (M), female (F), subadult (I) inhumations and 5 km, 5–20 km, 20–50 km baselines at Scalvinetto/Fondo Paviani. Red dots among baselines represent archaeological fauna samples.

The available baselines in the 5 km radius around the site range between 0.7096 and 0.7101 (Fig 10). Three out of four subadults match with the 5 km baselines, reinforcing the assumption that children are less likely to have migrated. The juvenile individual n. 352 showing a slightly lower ratio (0.7093) might have moved to the Scalvinetto/Fondo Paviani during late childhood or adolescence.

The majority of individuals show strontium isotope compositions that are compatible with the baselines within 5 and 20 km (0.7084–0.7101). Several individuals fall into the range of the broader hinterland (0.7072–0.7094), and four of them seem to be ‘outsiders’, namely burials n. 246, 205, 315 (adult females) and 405 (young-adult male). Their values, greater than 0.7122 up to 0.7196, are not consistent with any baseline values within the 50 km radius.

**Fig 11:**
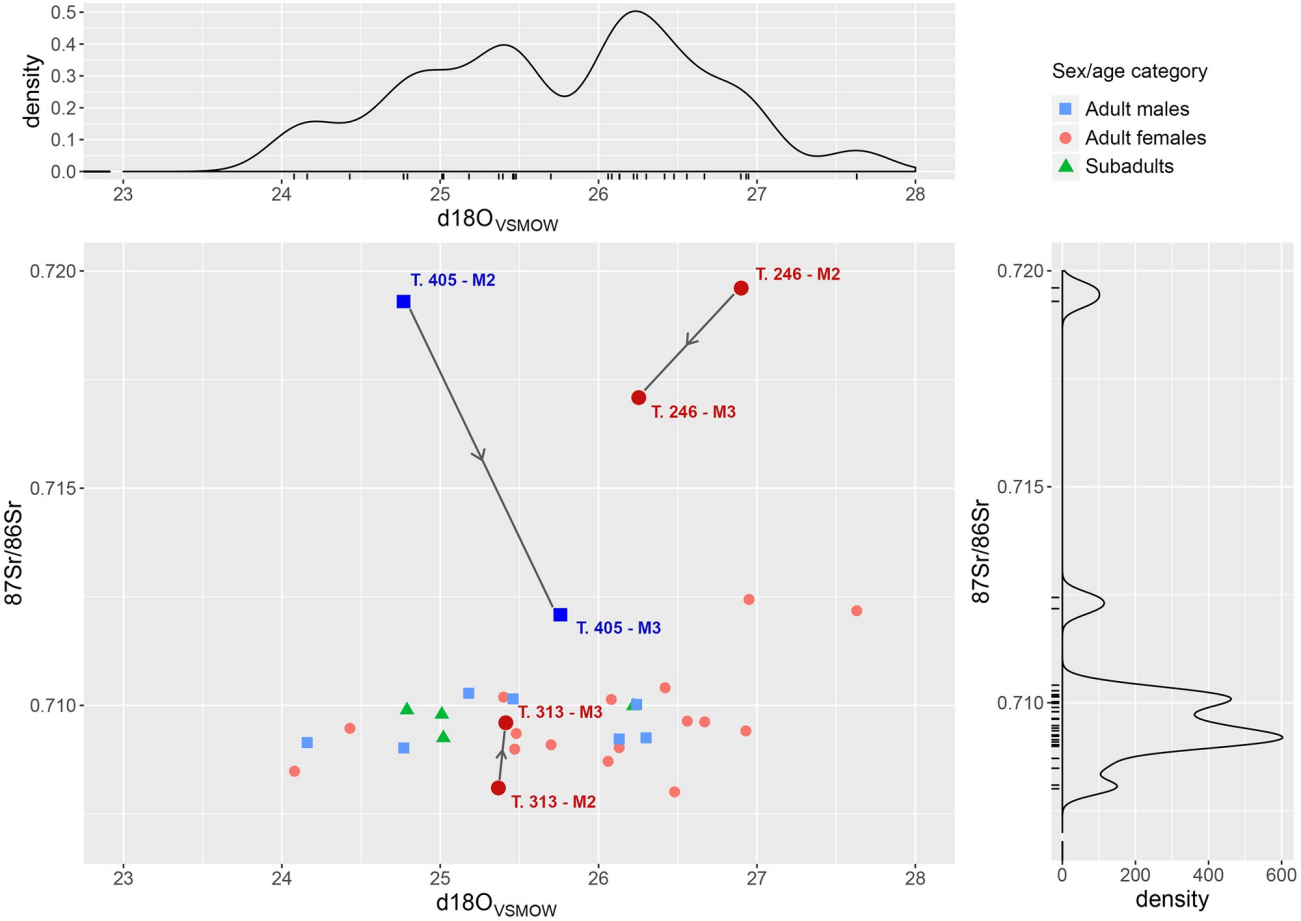
Scalvinetto/Fondo Paviani scatter and density plots of ^87^Sr/^86^Sr and δ^18^O_VSMOW (carbonate)_, with the indication of the sex/age categories. Three ‘life histories’ are highlighted: ^87^Sr/^86^Sr and δ^18^O_VSMOW_ were analysed in both second (M2) and third molars (M3) of three individuals: T. 246 (adult female), T. 313 (adult female), T. 405 (adult male).

δ^18^O values from carbonate in human tooth enamel range from 27.63‰ to 24.08‰ VSMOW, for a total range of 3.85‰, -almost twice as much as at Sant’Eurosia (Fig 11). The values cluster into two clearly separated groups, above and below 26‰. The lower group includes three (of four) subadults and approximates the value measured on animal enamel, 23.78‰, and might therefore reflect the ‘local’ oxygen isotopic composition. The second cluster, by contrast, seems to reveal the presence of people (mostly females) from warmer places and/or with different dietary habits that comprise a greater ingestion of processed (heated or fermented) water.

The conversion to the expected drinking water values [61] yields a range from -10.3‰ to -7.7‰ for the first ‘local’ cluster and from -7.2‰ to -4.7‰ for the second ‘non-local’ cluster. The ‘Local’ oxygen isotope ratio in today’s rainfall varies between -8‰ and -6‰ [49,50], Adige river waters between -12.3‰ and -10.8‰ [54] and Po river waters between -9.1‰ and -8.9‰ [53]; they support the idea that the first group is local, or from the hinterland of Fondo Paviani. The group with higher δ^18^O value seems instead composed of non-local individuals. Since more adult males and subadults are included into the ‘local’ group and more adult females among the ‘non-locals’, it might be suggested that Scalvinetto/Fondo Paviani community practice female exogamy to a significant extent.

**Fig 12:**
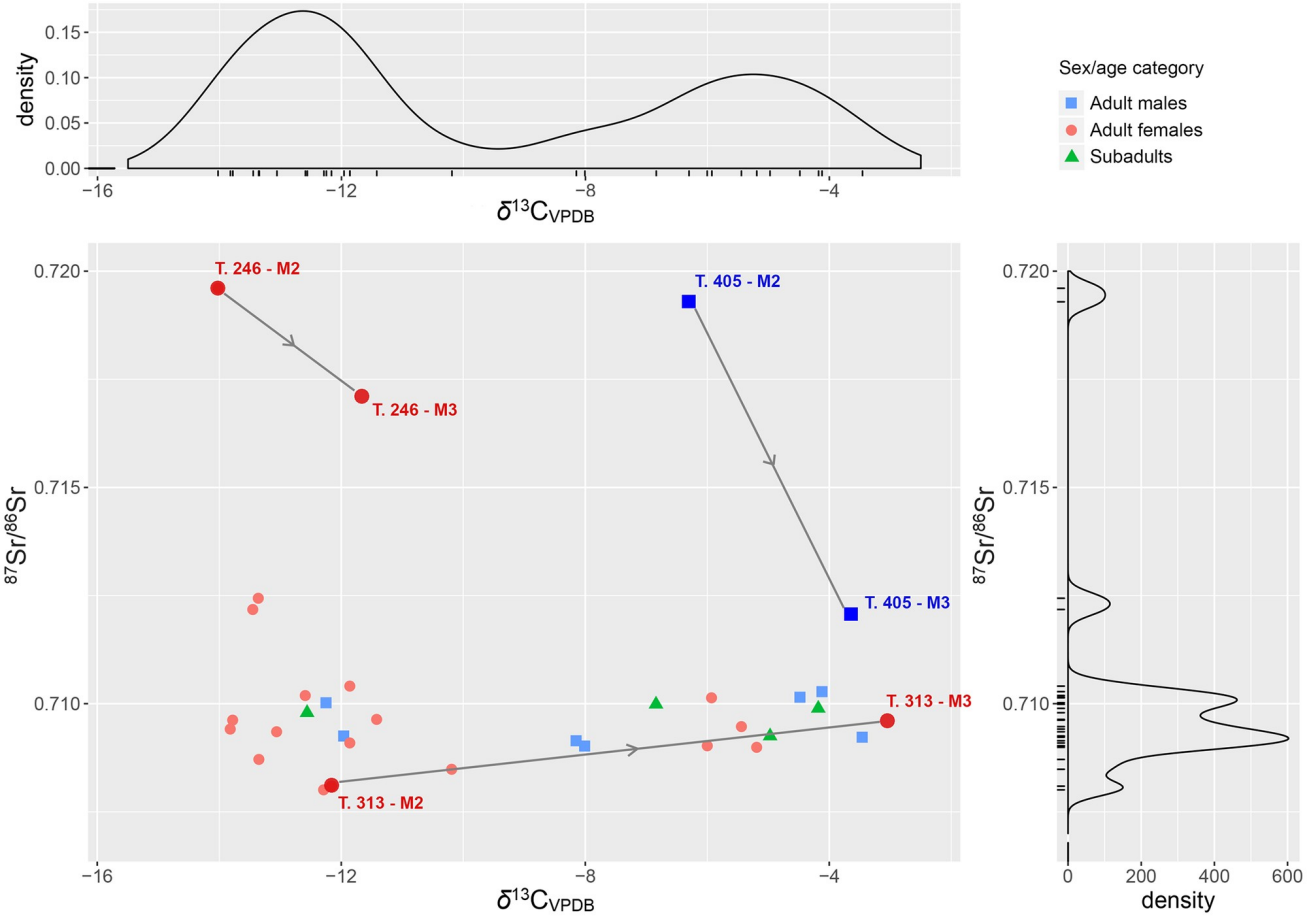
Scalvinetto/Fondo Paviani scatter and density plots of ^87^Sr/^86^Sr and δ^13^C_VPDB_, with the indication of the sex/age categories. Three ‘life histories’ are highlighted: ^87^Sr/^86^Sr and δ^13^C_VPDB_ were analysed in both second (M2) and third molars (M3) of three individuals: T. 246 (adult female), T. 313 (adult female), T. 405 (adult male).

The δ^13^C_VPDB_ values measured on carbonate of M2 tooth enamel once again show two clearly separated clusters of individuals: one, with values ranging from -14‰ to -10.2‰; the other from -8.2‰ to -3.5‰ (Fig 12). Pearson’s *r* coefficient, however, indicates that δ^13^C and δ^18^O covary only moderately (R = -0.51 [95% CI: -0.73 ‒ -0.18]).

According to Kellner and Schoeninger [115], the first cluster reflects a C_3_ protein/energy based diet with some supplement of C_4_, while the latter is clearly characterized by a C_3_ protein/C_4_ energy based diet. We cannot exclude the possibility that, since the analysed tooth is the M2, forming between 3 and 8 years of age, C_4_ food was introduced in the diet at a given age within this interval. However, taking into account the topographic distribution of burials. The individuals of the ‘C_3_ group’ are buried in the eastern part of the cemetery that include exculsively inhumations, rather densely distributed (S5 Fig). The burials of the ‘C_4_ group’, in contrast, spread in the northern and in the western parts of the burial area, occupied by later burials, as testified by the presence of cremations. Considering that cremations in the biritual cemeteries of the *Terramare* tend to be more recent than the inhumations, this spatial pattern might suggest that the C_4_ food was included in the diet in a middle/later phase of the cemetery. In the future, targetted radiocarbon dating may reveal the timing of introduction of C_4_ into the subsistence strategies of animals and humans at Fondo Paviani.

Scalvinetto’s first cluster values might reflect a range of approximately -19‰ to -15‰ on the collagen, while the second between -14‰ and -12‰. The values from the faunal remains (-2.8‰ on apatite, likely corresponding to roughly -9‰ on collagen), nonetheless, suggest that some animals (pigs) at Fondo Paviani were probably fed with C4 plants or had a diet similar to the humans, as also confirmed in recent work by Tafuri et al. [65].

The high δ^13^C in the collagen of individuals and animals from the closely situated *terramare* at Olmo di Nogara, Vallona di Ostiglia, Bovolone and Fondo Paviani suggests that the use of millet as fodder was rather common during the Middle-Late Bronze Age in Northern Italy [65,118,119]. As millet is characterized by a short growing season and a high yield and is quite adaptable to dry environment and soils, it may have been a more suitable crop in the phase of drought that probably occurred in the final phase of the *Terramare* period, also aggravated by the heavy human impact on vegetation until 1100 BC, which led to a wide deforestation (up to 80% open land) of the cultivable terrain [120,121].

Through the analysis of the M3 enamel in three individuals (T. 246, T. 313, T. 405), we can try to identify in which phase of their life the movement occurred and if there was any change in dietary habit between childhood and adolescence (Figs 11 and 12). The adult female T. 313 probably reached Fondo Paviani during her adolescence, as the M2 ^87^Sr/^86^Sr suggests a non-local origin (compatible with the 20–50 km baseline), whilst the M3 value instead falls within the ^87^Sr/^86^Sr local baseline. Her δ^18^O does not significantly vary, but a radical shift from C_3_- to C_4_-diet (directly or indirectly from animals) is indicated by the shift of +8‰ in δ^13^C_VPDB_.

The adult male T. 405 shows a high strontium isotope ratio in the M2 and a much closer value to Fondo Paviani local baselines in the M3, which probably indicates his displacement from the Alpine valleys (i.e. west of the Lake Garda, or along the upper course of the Adige River) during an undefined period between childhood and late adolescence. His oxygen isotope composition, however, does not indicate the intake of the cold waters of Alpine rivers, but seems to reflect the rainfall δ^18^O of the mountain valleys [50,54]. Interestingly, his carbon isotope ratios show instead a progressively higher consumption of C_4_ food between childhood and adolescence.

The adult female T. 246 has an ^87^Sr/^86^Sr similar to T. 405 in the M2, whereas the M3 value lies far off the local ratios at Fondo Paviani. The high δ^18^O values indicate that T. 246 was probably born in a distant place and reached Fondo Paviani at a later stage of her adolescence or during early adulthood. Her δ^13^C values fall within the range for a C_3_ based diets, though the shift of +2‰ might indicate an increased consumption of C_4_-foods during the formation of the M3.

### Scalvinetto cremations

The range shown by subadults (0.7096–0.7100) is consistent with the 5 km baselines (0.7096–0.7101), as expected.

**Fig 13:**
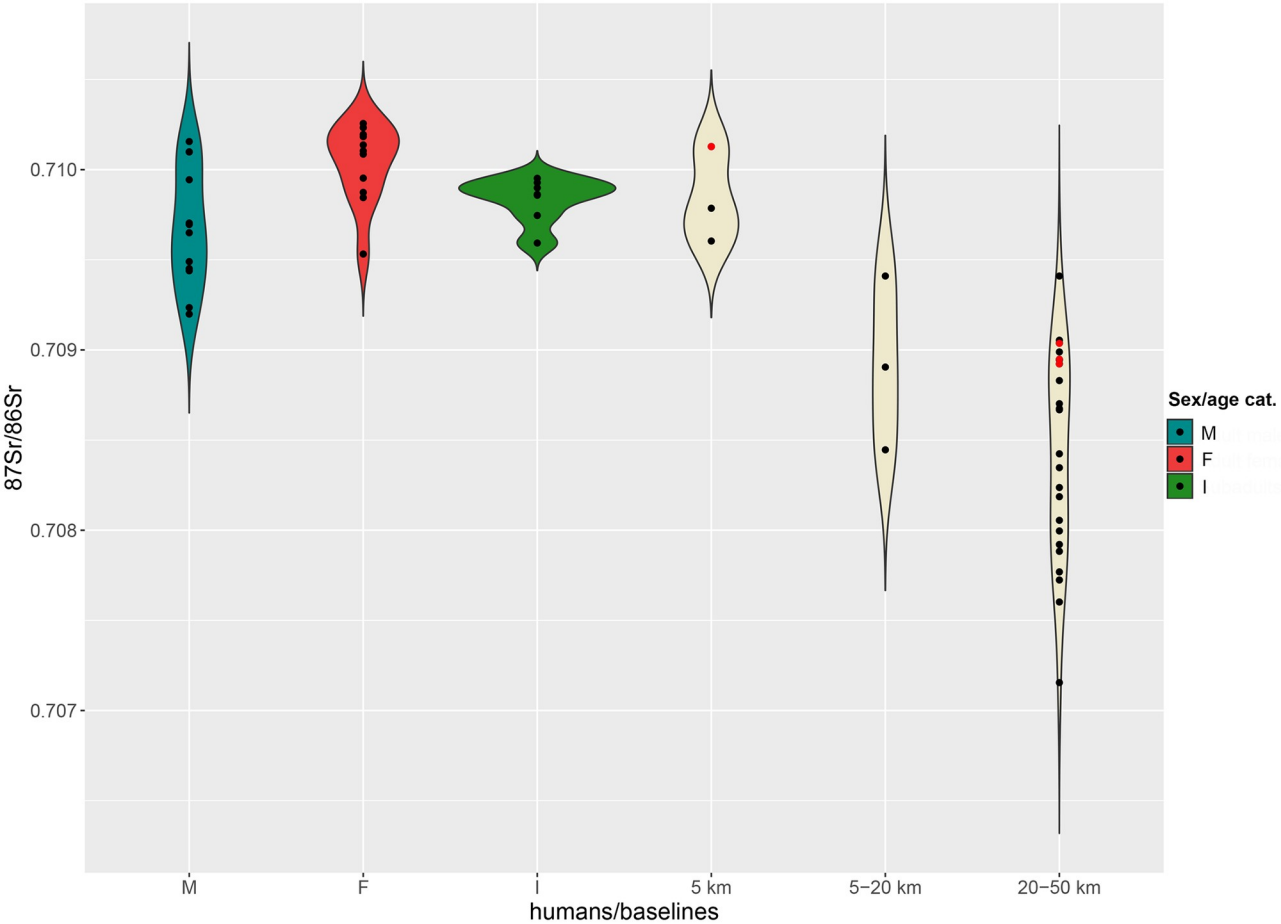
Violin plot. Distribution of the ^87^Sr/^86^Sr values from adult males (M), females (F), subadults (I) cremations and 5 km and 5–20 km, 20–50 km baselines at Scalvinetto/Fondo Paviani. Red dots among baselines represent archaeological fauna samples.

The 12 adult males range between 0.7092 and 0.7102; six of them are compatible with the 5 km radius baseline, five with the 5–20 and 20–50 km baselines, and one (T. 474) might be an outlier, coming from a place further than 50 km, although its value is slightly higher than the 5 km baselines. Compared to males, the 11 adult females display a narrower range, between 0.7095 and 0.7103. The violin plot (Fig 13) shows that six females (between 0.7096 and 0.7101) are compatible with the 5 km radius baseline: one is compatible with the 5–20 and 20–50 km baselines, and four are characterized by values slightly greater than the 5 km baselines (>0.7101). These latter are compatible with the isotopic baseline of the Brenta River valley and/or middle Adige River valley, both located further than 50 km from Scalvinetto/Fondo Paviani. Interestingly, among the analysed individuals there is no trace of females born in the close or broader hinterland (5–50 km from the site), while a significant number of might be true ‘outsiders’ from further distance, probably from the same area.

## Discussion

The main results of the present study can be summarized as follows:

1. *Strontium isotope baselines*. The northern and southern parts of the Po river valley show a different amplitude of bioavailable ^87^Sr/^86^Sr baselines. In the southern district, where Sant’Eurosia and Casinalbo are located, the range is significantly narrower both in the plain and in the mountains. Therefore, human mobility within that area and across short distances is harder to retrace, while movements over a broader radius, especially from the northern side of the plain, are more easily identifiable. In the north, where Scalvinetto/Fondo Paviani is situated, the range of ^87^Sr/^86^Sr baselines is much broader, as consequence of the local and regional variety of geolithological units and hydrogeological basins forming the alluvial plain. For these reasons, the trajectories of individual human movements within the northern basin of the Po valley can be suggested with greater precision, although, for the same reasons, we must be aware of the risk of identifying as people from Northern Italy individuals from other, more distant, areas with the same isotopic signature.
  As previously noted by Emery et al. [21], who published a first preliminary ‘isoscape’ of bioavailable ^87^Sr/^86^Sr variation across Italy, more baselines are required to further refine the map. Indeed, some of the new bioavailable strontium isotope ratios provided here do not interpolate Emery et al.’s isoscape of the Po and Adige basins, while others fit well. There are, obviously, gaps in the current maps that future isotope studies will need to fill.
  In evaluating the different sources of strontium used for baselines, we can, for example, compare the values obtained for Emilian Pliocene/Pleistocene limestone: the bedrock yielded a mean ^87^Sr/^86^Sr of 0.7087, soils 0.7087, snail shell 0.7085, springwater 0.7088, and wine 0.7090. Similarly geolithological zones 1, 2, 7–9 all display narrow ranges from a variety of samples and lithologies. We can therefore conclude that even if there are slight variations of the isotopic composition, these are nonetheless relatively small, and the ultimate impact of anthropogenic strontium (fertilizer/pollution) is negligible. Confirmation seems to come from the ^87^Sr/^86^Sr values of children (most probably not immigrants) and animals at Sant’Eurosia and Casinalbo, which are both related to same Cenozoic carbonates and the alluvial plain derived from them. At Sant’Eurosia children range between 0.7089 and 0.7090 and domestic animals between 0.7082 and 0.7092; at Casinalbo children range between 0.7085 and 0.7090 and domestic animals between 0.7087 and 0.7089. Modern grapes from Parma are close to 0.7087. In both cases, the two archaeological sample types are largely consistent with the other baseline sources and therefore strengthen and validate the baseline based on modern materials. Additional sources for local baselines are nonetheless necessary to refine this preliminary framework.
2. *Oxygen and carbon isotopes*. Adding oxygen isotope analysis to strontium, in the case of inhumations, has represented a useful tool for distinguishing ‘outsiders’, whose origin from a place with a similar ^87^Sr/^86^Sr baseline tended to hide them among the locals. The use of δ^18^O ‘isoscapes’ based on modern rainfall and ground-waters is not, however, straightforward, as many factors can intervene to change the ratio of drinking water. The examination of faunal remains and of the distribution of the individual values can be complementary in assessing the range of the local baseline. Since the analysis of carbonates for δ^18^O in tooth enamel also simultaneously determines δ^13^C, this approach can also be informative about diet, in terms of C_3_/C_4_ protein/energy consumption. δ^13^C analysis is of particular interest for phases such as the Middle/Late Bronze Age in Northern Italy, when millet was introduced as fodder for animals and possibly also into the human diet [65,118,119].
3. *Strontium isotopes on cremations*. The ^87^Sr/^86^Sr values measured on the petrous bone and M2 tooth enamel of the same individual from Casinalbo show a difference of 0.00005, thus providing additional confirmation of the reliability of strontium isotope analysis on cremated petrous portion and encouraging further applications [43,122]. A similar test has recently been conducted on two cremated individuals from the Late Bronze Age site of Le Narde di Frattesina, resulting in the same correspondence between tooth enamel and petrous portion values.
  As oxygen isotope analysis is not a viable option for cremations, the use of lead isotopes could eventually add a supplementary parameter for evaluating mobility [52], but currently this approach suffers from a lack of isoscape data and has not been empirically validated for cremated bone. At the current stage of the research, however, we may argue that in the biritual cemetery of Scalvinetto movements of people occurred more frequently and over a broader radius among those inhumed rather than cremated. However, more mobility studies are needed for a better understanding of the transition from the practice of inhumation to the ‘urnfield model’ around the 15^th^ century BC in Northern Italy.
4. *Human mobility*. The Early Bronze Age population buried at Sant’Eurosia might be regarded as rather mobile, as the strontium and oxygen isotope ratios are dispersed. The related settlement is still unknown, like the vast majority of Early Bronze Age settlements (with the exception of lake-dwellings), since they were probably relatively impermanent [123]. Interpreting mobility might therefore take into account different possibilities.

**Fig 14:**
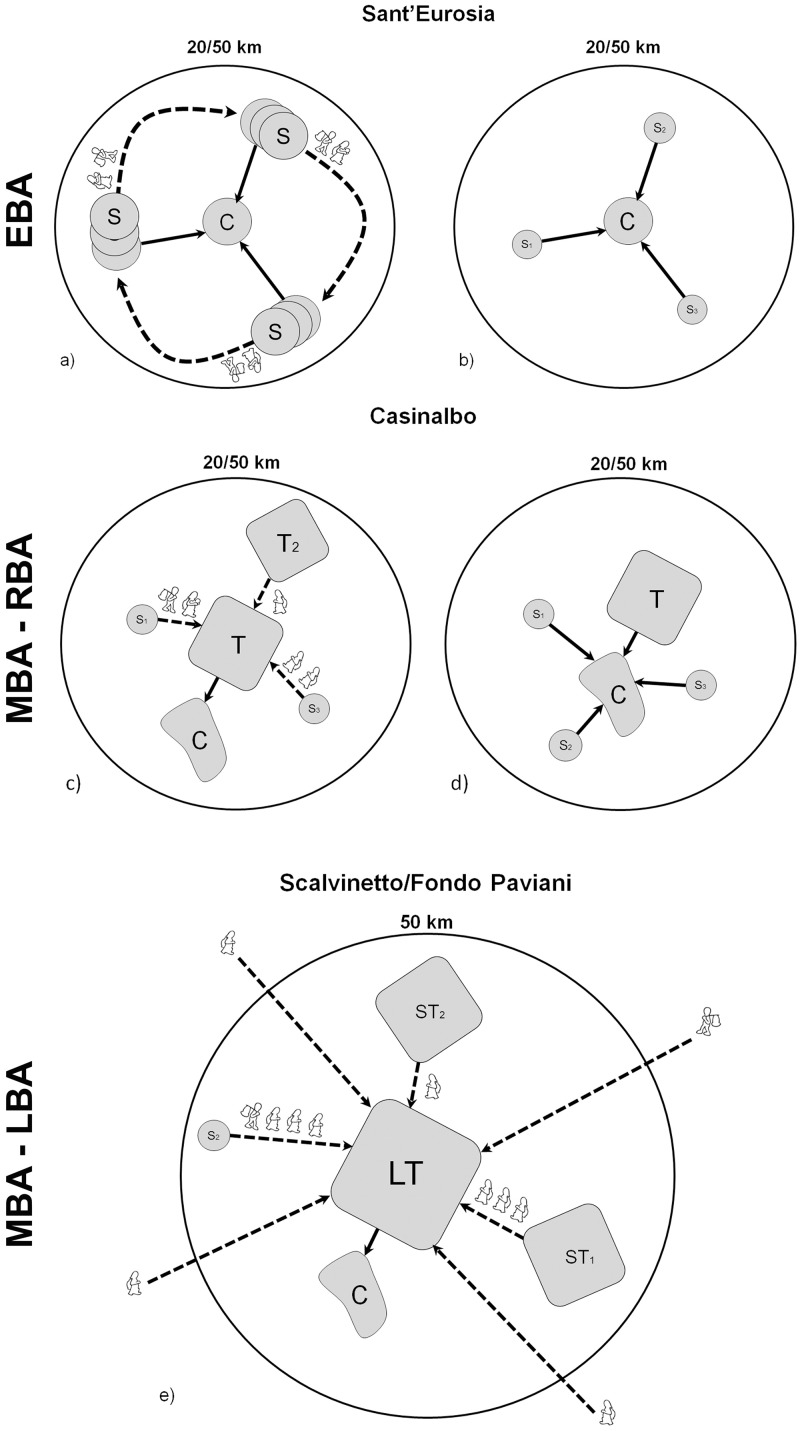
Different models of mobility for the three investigated sites. Sant’Eurosia and Casinalbo might be interpreted at least in two different ways (S = settlement; S1,2… = settlement 1, settlement 2…; C = cemetery; T = *terramara*; LT = large *terramara*; ST1,2… = small *terramara* 1, small *terramara* 2).

Sant’Eurosia, case ‘a)’ (Fig 14). In spite of the evidence of its collective mobility, the community persisted in burying their dead in the same place. No significant differences can be observed between the mobility patterns of males and females, whose origin seems largely identifiable at the site and in the hinterland area, although a small number of males might have come from north of the Po River, or from the upper Taro river Valley (geolithological zone 3). The carbon isotopes on apatite nonetheless indicates that some kind of change occurred between the social segment buried under the tumuli (generally older) and the burial group in the (generally more recent) flat graves. This discrepancy between the two groups could be related to a shift in the settlement location or of the fields where food was produced, a change in the diet/subsistence strategy, a change in climatic conditions, or a combination of factors.

Sant’Eurosia, case ‘b)’ (Fig 14). An alternative interpretation sees Sant’Eurosia as a monumental burial area where different households and/or small settlement groupings spread over a relatively limited territory, used different tumuli or groups of flat graves according to their group membership. However, this hypothesis seems less likely, since individuals buried under the same tumulus do not show homogeneous isotopic compositions. Further analyses (^14^C, dietary isotopes, aDNA) might reveal whether the population buried at Sant’Eurosia is representative of the whole community or of a more restricted hegemonic group.

The Middle and Recent Bronze Age urnfield of the *terramara* at Casinalbo shows a different pattern of mobility. The concentration of males in a very narrow ^87^Sr/^86^Sr interval and the dispersion of females appears to suggest a high degree of patrilocality and the occurrence of exogamic practices within the immediate and/or the broader hinterland radius (Fig 14, Casinalbo case ‘c)’), a tendency that has been observed in other European Bronze Age studies, combining isotopes and mtDNA [124]. Interestingly, adult females with remoter origins are normally also those with richer grave goods.

The fact that some children show values compatible with the hinterland might be explained in terms of displacements during the early stages of life. Although difficult to demonstrate, we might also suggest that children were part of fosterage practices for reinforcing alliances, kinship ties, and mutual trust in order to prevent eventual conflicts.

Another possibility, although less probable, is that they spent their life in satellite centres of the immediate hinterland, and were only buried in Casinalbo urnfield, which, in that case, would represent a cemetery, serving not just the *terramara* itself, but also other nearby settlements (Fig 14, Casinalbo case ‘d)’).

Only one adult male seems to have an origin in the broader hinterland, and one from an even further radius. The Casinalbo community therefore appears well-established in the local territory, with marriage customs (and perhaps fosterage practices) probably crucial for the creation and maintenance of socio-political relationships/alliances with the neighbouring communities. Considering that the site and area in general have been deeply investigated from an archaeological point of view, further isotopic investigations could produce an extremely refined picture of the dynamics of mobility on the local scale.

From the Middle and Late Bronze Age biritual cemetery at Scalvinetto (*terramara* at Fondo Paviani) we are able to reconstruct a much more articulated framework (Fig 14, Scalvinetto/Fondo Paviani case ‘e’). Our results, considering both strontium and oxygen isotope composition on inhumations and only strontium on cremations, suggest that 28 out of 60 individuals (47%) are not indigenous. Remarkably high ^87^Sr/^86^Sr values (>0.7110) are documented exclusively among inhumations. Regarding cremations, 19 individuals are compatible with the site baseline, 6 with the 5–20 km and 20–50 km baselines, and 5 (mostly females) possibly with further distances, though not the Alpine areas (geolithological zone 10). Concerning inhumations, the analysis of δ^18^O has allowed us to discriminate a considerable number of non-indigenous persons among those individuals that fall in the 5 km ^87^Sr/^86^Sr baseline. This also means that the number of outsiders could have been underestimated among the cremations. Among the inhumations, 17 individuals have been identified as non-indigenous. According to the ^87^Sr/^86^Sr results, the non-indigenous individuals show a wide spectrum of different provenances, basically from all the geolithological zones. These data reinforce the idea that the *terramara* at Fondo Paviani, as a consequence of its status as a ‘central place’, attracted a large number of people from different places, both from the hinterland (intra-polity networks) and from a broader radius (inter-polity networks). The occurrence of locally produced Apennine, Aegean-Mycenaean and Levantine-Cypriot Bichrome style ceramics might reflect the presence of foreign potters [125,126], although Jung has considered the possibility that Italian artisans moved (or were sent) to Greece to acquire the know-how from the local ceramic specialists and came back following an apprenticeship [127,128].

Similarly to Casinalbo, females appear to have been highly mobile, and may come from the immediate or broader hinterland, as well as further distances, especially from warmer places, to judge by the cluster with δ^18^O greater than 26‰. In contrast to Casinalbo, males are equally mobile. The simplest explanation seems to lie again in marriage practices, but other options cannot be discounted. Subadults, with only one exception, all seem indigenous, confirming that the analysis of younger individuals may be used to strengthen the ‘local’ isotopic signatures.

δ^13^C data on the enamel bioapatite have highlighted the presence of a group of individuals (as well as one animal), characterized by a substantial component of C_4_ plants (millet) in the diet at the age of 3–8 years of age, when the analysed teeth were forming, and another group with a C_3_ plant oriented regime. Further stable isotope analysis on bone collagen, and possibly on M3 apatite, as well as radiocarbon dates, might contribute to clarify the times and modes of millet introduction among the *terramare* [65], and/or about the phase of life when millet became an essential part of people’s diet.

Looking at the more general picture, we can conclude that mobility patterns in the Po plain were strictly entwined to the social structures and political strategies of the communities.

Despite the limited information about the related settlements, the model for the Early Bronze Age seems to confirm the idea of small communities, mobile within the hinterland across the generations, as they shifted to different areas of exploitable land.

With the progressive stabilization of settlements, within the flourishing of the *Terramare* system (Middle and Recent Bronze Age, 1650–1150 BC), mobility ceased to involve the residential community as a whole, but only specific categories of individuals (mainly women), whose displacement was probably tied to alliances and negotiations across surroundings and distant areas.

However, tied to the Late Bronze Age tendency to strengthen the hierarchical organization of polities, clear differences emerged between relatively small settlements interacting on a more ‘local’ scale, and large nodal cores of the systems in the plain intensively involved in a wider circulation of goods and people within the hinterland, but also far beyond local and regional boundaries. The strength of this ‘inequality of mobility’ is striking.

In this phase of economic and demographic growth, the dynamism and the permeability to outsiders of the large centres was probably one of the factors that determined their (temporary) socio-economic prosperity. The power of attraction exercised by nodes was probably underpinned by traditional marriage practices, but also represented a response to a demand for labour required by the huge infrastructural works and by the specialized craftsmanship for the development locally of an industry imitating powerful exotic commodities. It can be assumed that a high degree of mobility and permeability was demographically and socially sustainable up to a certain threshold and that the role played by all these movements in the subsequent crisis of the *Terramare* system (as well as by other political entities in the Mediterranean) must be taken into consideration.

The collapse of the *Terramare* at the end of the Recent Bronze Age (first half of the 12^th^ century BC) likely caused a contraction of networks of mobility and a redefinition of power relations [11]. This might probably be reflected in new trajectories, scales and forms of mobility for the communities settled in Northern Italy, which were engaged in the attempt of organizing political centralization and institutionalisation that became one of the characteristic traits of Iron Age societies.

## Conclusions

Our results provide the first isotopic framework of human mobility in the 2^nd^ millennium BC of Northern Italy; a picture that further analyses, above all aDNA, can fruitfully build upon.

This study of 104 individuals in three key-burial sites has allowed us to observe the variations of mobility patterns throughout the different phases of the Bronze Age. Since the Middle Bronze Age, as a consequence of the stabilization of settlements, mobility became part of socio-political negotiations and began to involve preferentially women, in a system that appears undoubtedly patrilocal. Clear differences arose between relatively small settlements more active on the ‘local’ scale, and large centres interacting with the hinterland, but also far beyond regional boundaries.

Furthermore, the results of a test carried out on ^87^Sr/^86^Sr obtained on cremated petrous portion and second molar encourages further applications of the isotopic analyses on cremated remains.

The analysis oxygen and carbon isotope ratios in inhumed populations also allowed to us to discriminate more groups of non-indigenous people and to identify at Scalvinetto/Fondo Paviani a significant number of individuals, characterised by the consumption of C_4_-food (probably millet).

## Supporting information

S1 Table Database of ^87^Sr/^86^Sr baselines for the investigated area. (XLSX)

S2 Table Geolithological zones and variability of bioavailable ^87^Sr/^86^Sr baselines. (XLSX)

S1 Fig BASr isoscape baseline values for the investigated area. Numbers mark the geolithological zones in S1 and S2 Tables. The map is constructed by using public domain wms data downloadable from http://wms.pcn.minambiente.it/ogc?map=/ms_ogc/WMS_v1.3/Vettoriali/Carta_geolitologica.map under a CC BY license, with permission from Geoportale Nazionale and plotting data from S1 Table. Stars, dots and pentagons indicate the ^87^Sr/^86^Sr baseline samples (stars = animals and plants; dots = spring waters; pentagons = Po river waters).
(TIF)

S2 Fig EBA tumuli at Sant’Eurosia and different types of burials: Central burial (left) and ditch burials (center) under the tumuli; flat graves outside of the tumuli (right) with the two skeletons in the head-to-toe position (mod. after [84]). (TIF)

S3 Fig Map of Casinalbo urnfield (mod. after [12]). (TIF)

S4 Fig Map of Scalvinetto biritual cemetery. Inhumations are more frequent in the western and northern part of the burial area, while cremations in the eastern sector. In the frame a detail of the area where the two type of burial overlap (mod. after [94]).
(TIF)

S5 Fig Map of Scalvinetto/Fondo Paviani cemetery. ● = lower δ^13^C_VPDB_ values, ● = greater δ^13^C_VPDB_ values, ● = cremations. (TIF)
