# Supplementary figures and images for: Flows of people in villages and large centres in Bronze Age Italy through strontium and oxygen isotopes
Source: PLoS One. 2019 Jan 9;14(1):e0209693. doi: 10.1371/journal.pone.0209693 (PMC6326466; doi:10.1371/journal.pone.0209693)

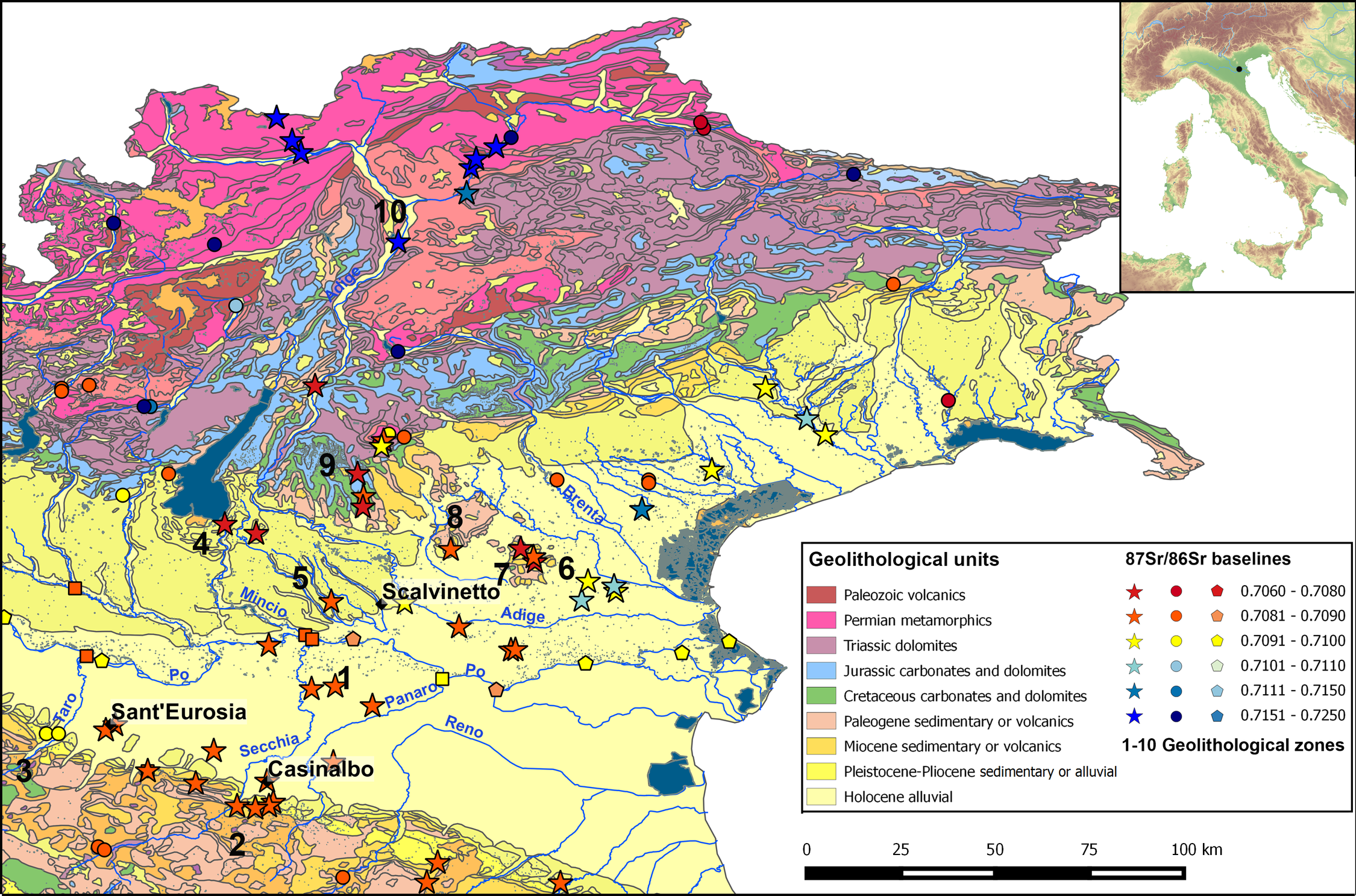

Supplement: S1 Fig — Numbers mark the geolithological zones in S1 and S2 Tables. The map is constructed by using public domain wms data downloadable from http://wms.pcn.minambiente.it/ogc?map=/ms_ogc/WMS_v1.3/Vettoriali/Carta_geolitologica.map under a CC BY license, with permission from Geoportale Nazionale and plotting data from S1 Table. Stars, dots and pentagons indicate the 87Sr/86Sr baseline samples (stars = animals and plants; dots = spring waters; pentagons = Po river waters). (TIF) [file pone.0209693.s003.tif]

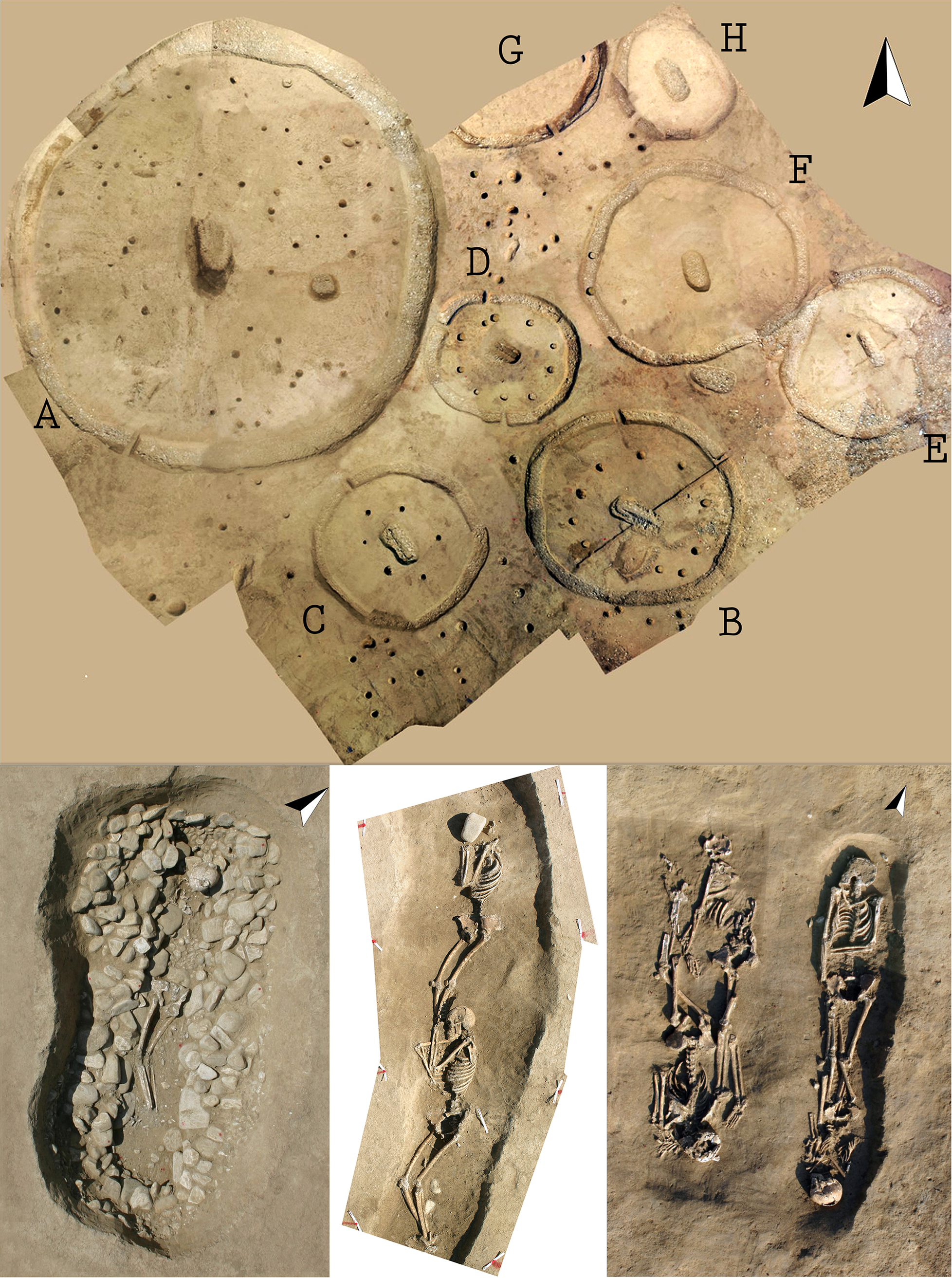

Supplement: S2 Fig — (TIF) [file pone.0209693.s004.tif]

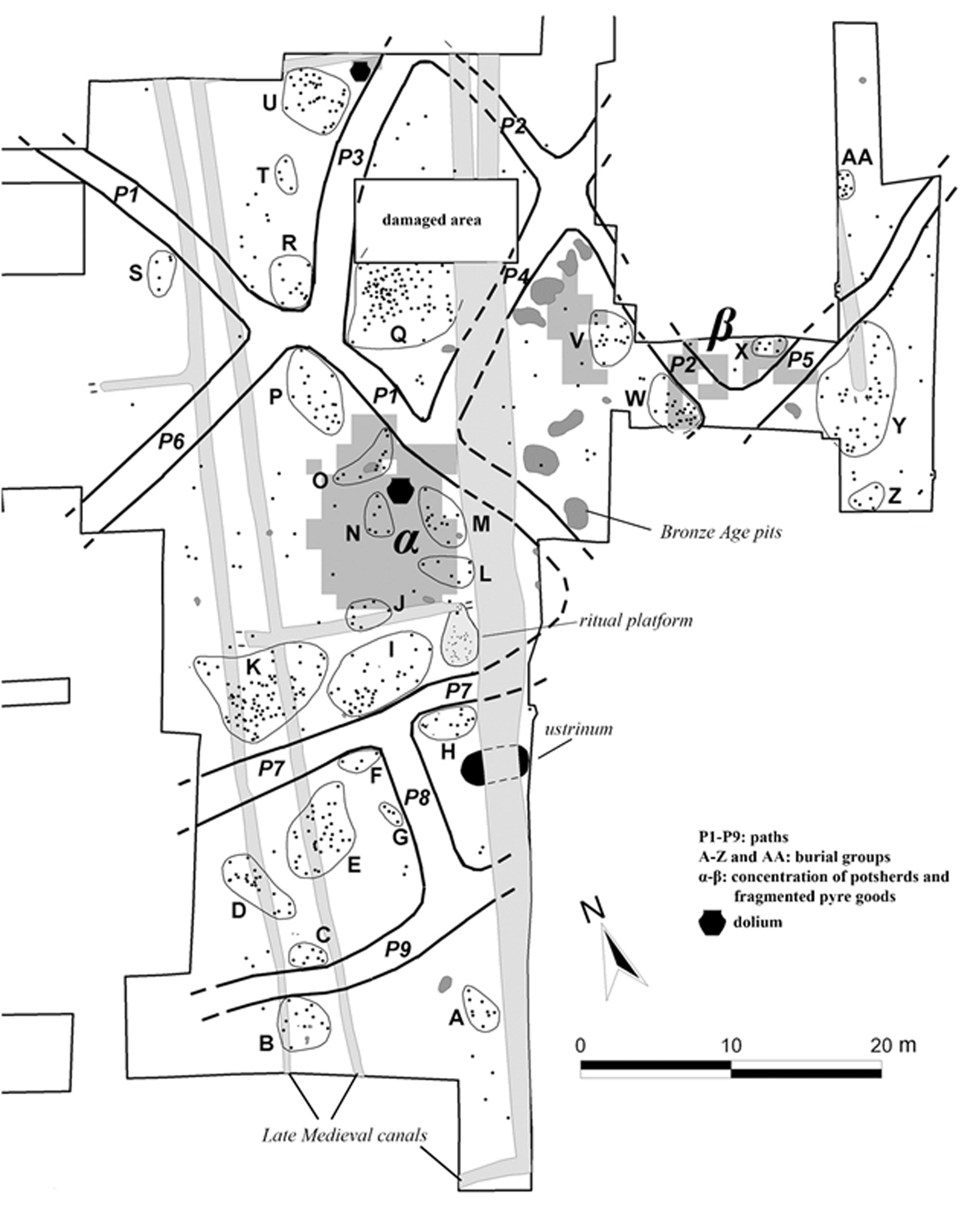

Supplement: S3 Fig — (TIF) [file pone.0209693.s005.tif]

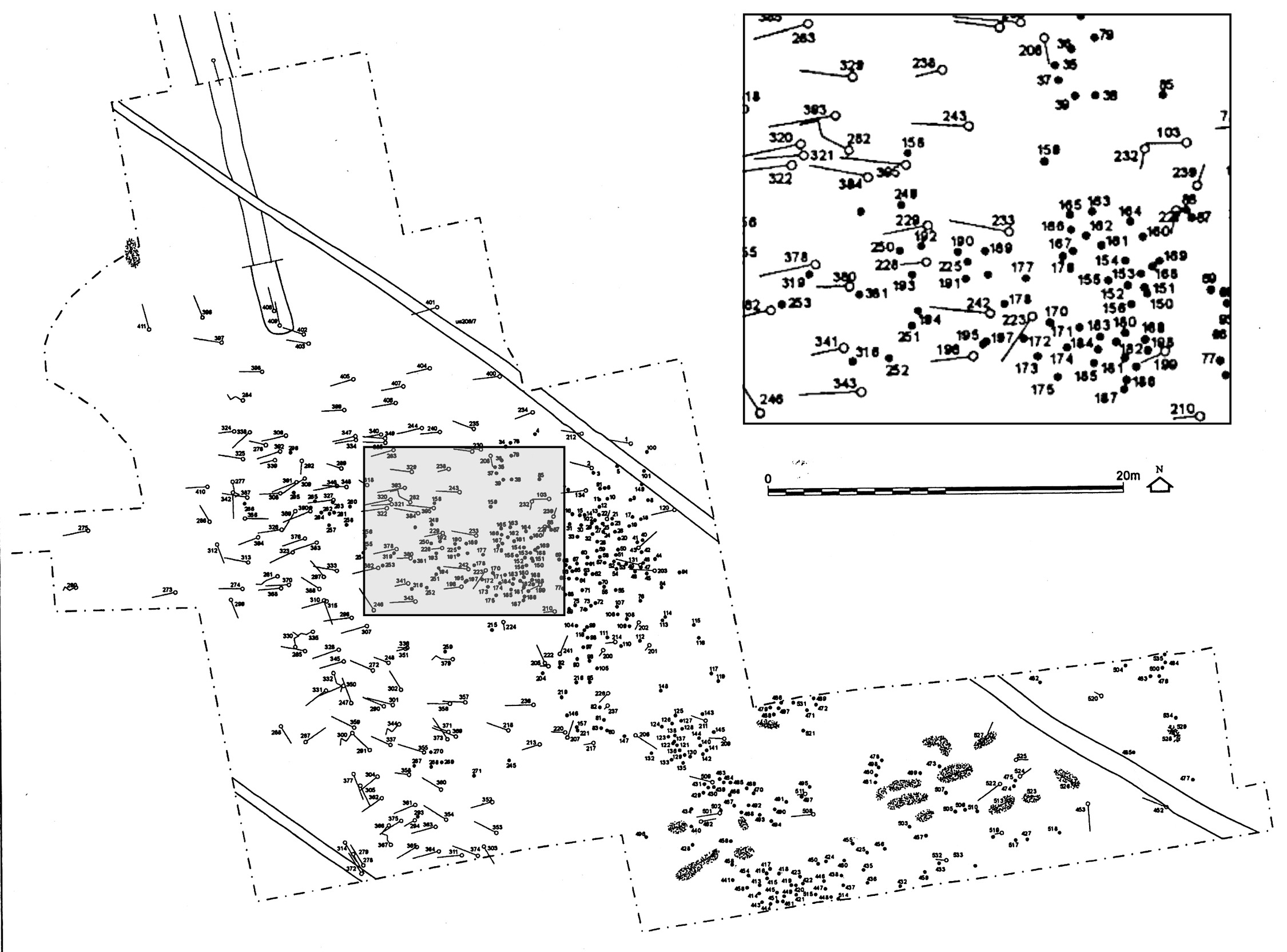

Supplement: S4 Fig — Inhumations are more frequent in the western and northern part of the burial area, while cremations in the eastern sector. In the frame a detail of the area where the two type of burial overlap (mod. after [94]). (TIF) [file pone.0209693.s006.tif]

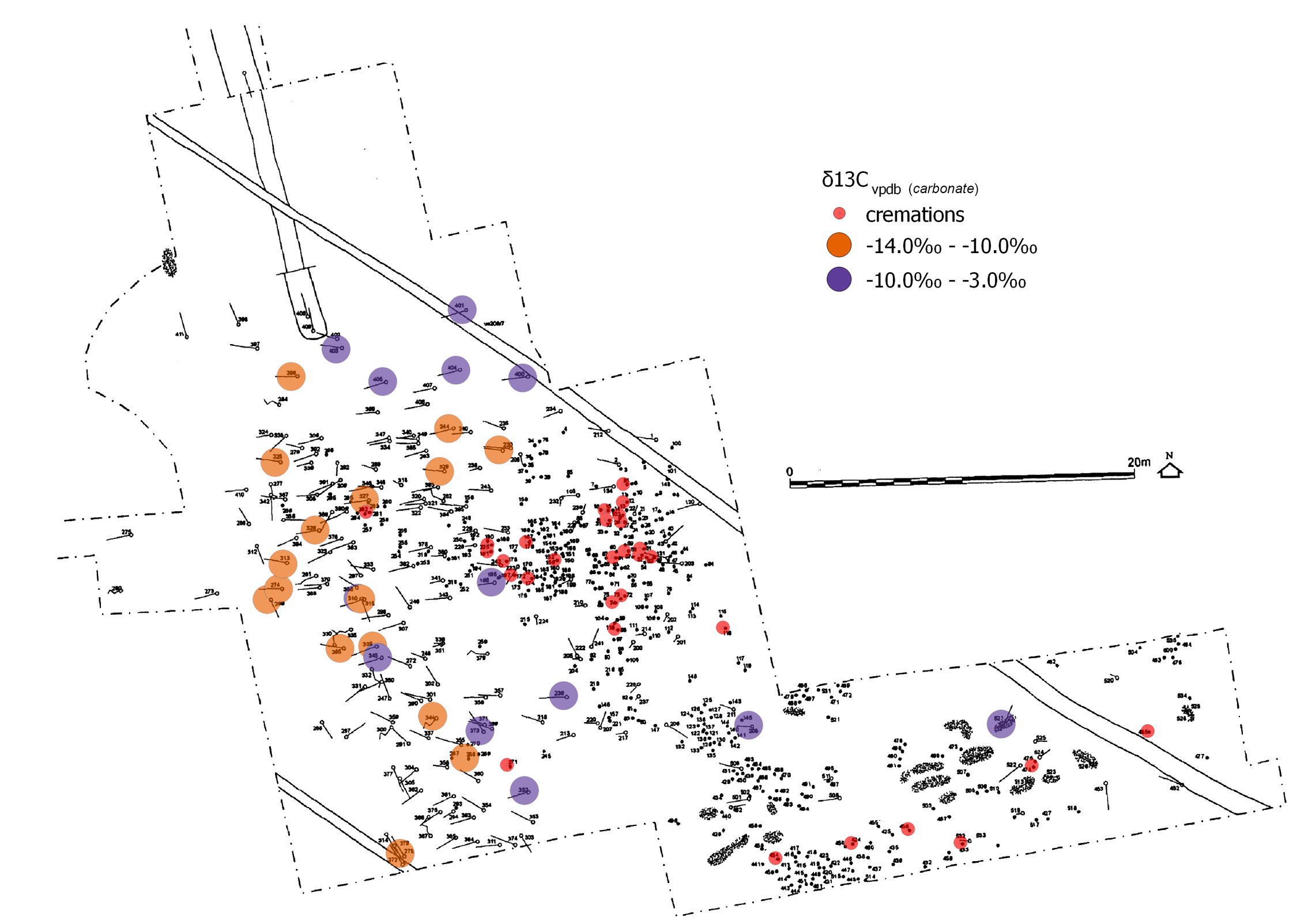

Supplement: S5 Fig — (TIF) [file pone.0209693.s007.tif]
